# Portable electrochemical sensing coupled with machine learning for rapid Arabica and Robusta coffee authentication and blend ratio prediction

**DOI:** 10.1016/j.fochx.2026.104523

**Published:** 2026-09-26

**Authors:** Bing-Chen Gu, Kuan-Jung Chung, Zhen-Wei Hong, Ning-Hsieh Yang, Chia-Che Wu

**Affiliations:** a Graduate Program in Semiconductor and Green Technology, Academy of Circular Economy, National Chung Hsing University, Nantou County, Taiwan; b Department of Mechatronics Engineering, National Changhua University of Education, Changhua, Taiwan; c Department of Mechanical Engineering, National Chung Hsing University, Taichung, Taiwan; d i-Center for Advanced Science and Technology, National Chung Hsing University, Taichung, Taiwan

**Keywords:** Electrochemical sensing, Machine learning, Arabica, Robusta, Blend ratio prediction

## Abstract

In this study, we developed a portable electrochemical sensing platform integrated with machine learning for the rapid identification of coffee species and quantitative prediction of blend ratios. Electrochemical measurements targeting caffeine, chlorogenic acids, trigonelline, and reducing sugars were performed and the roast level was incorporated as an additional analytical feature. The resulting electrochemical fingerprints captured both species-dependent compositional differences and roasting-induced chemical transformations. Using these features, an extreme gradient boosting classification model achieved high accuracy in distinguishing arabica, robusta, and blended coffee samples. For blend-ratio regression, the random-split ANN result (R^2^ = 0.980 and RMSE = 0.050) was retained as a within-distribution benchmark. Under nested group-aware leave-one-blend-pair-out validation, the ANN ensemble achieved R^2^ = 0.9121, RMSE = 0.0676, and MAE = 0.0547, with 87.0% of predictions within ±0.10 of the reference blend fraction. External validation using literature-reported compositional datasets provided complementary support of species identification. Compared with conventional analytical methods, the proposed electrochemical–ML framework requires only a small aliquot of coffee extract, and the electrochemical measurement can be completed within 3 min after sample preparation.

## 1 Introduction

Global coffee production is dominated by two species: *Coffea Arabica* (Arabica) and *Coffea canephora* (Robusta). Arabica is typically cultivated at elevations above 600 m, with major production in Latin America, East Africa, and India. Owing to its lower perceived bitterness and more desirable flavor profile, Arabica is preferred in specialty coffee markets. Generally, it commands a price more than twice that of Robusta [Sahachairungrueng et al., 2022]. Meanwhile, Robusta is grown at lower altitudes, predominantly in West Africa, Indonesia, Vietnam, and Brazil. It is often characterized by earthy, woody, and smoky flavor notes, along with lower acidity and a heavier body. Its higher caffeine (CAF) and lipid contents also make it valuable in espresso blends, where modest additions can enhance crema formation. Robusta is frequently used in commercial blends to balance flavor attributes and reduce production costs, with blend ratios tailored to specific beverage styles [Wang et al., 2019]. For example, maintaining the Robusta proportion below 20% can enhance flavor complexity without substantially diminishing key aromatic compounds while reducing costs by ∼5%–20%. Higher Robusta proportions (≈40%) typically increase bitterness and body weight and may be preferred for beverages such as Americano and cold brew, with cost reductions of ∼25%–35%. At still higher proportions (≈60%), the beverage becomes markedly more bitter and stimulating, which may be suitable for instant or flavored coffee products, with potential cost savings of up to ∼45% [Kalschne et al., 2019; Abubakar et al., 2020]. Previous studies further indicate that steamed defective *C. canephora* can be incorporated into Arabica-based blends while maintaining characteristic sensory attributes and acceptable consumer responses [Kalschne et al., 2019]. Nevertheless, the substantial price disparity between the two species has encouraged adulteration, in which products marketed as “100% Arabica” are blended with cheaper Robusta or other materials for economic gain. Although green coffee beans can often be distinguished visually, their morphological characteristics largely disappear after roasting and grinding, making chemical authentication necessary for reliable verification [de Carvalho Couto et al., 2023].

Recent research has increasingly examined the relationships among coffee flavor, varietal characteristics, and chemical composition. Ky et al. [2001] used high-performance liquid chromatography (HPLC) to quantify CAF, trigonelline (TG), chlorogenic acids (CGAs), and sugars in Arabica and Robusta beans and reported higher TG and sugar concentrations in Arabica, whereas Robusta contained higher CAF and CGA concentrations. Farah et al. [2006] compared the chemical composition of graded Brazilian coffees and observed positive associations between coffee quality and TG and CAF concentrations. Meanwhile, lower-grade coffees (including Robusta) tended to exhibit higher CGA concentrations. Ogutu et al. [2022] reported that cultivated Arabica exhibited higher TG and sugar levels but lower CAF and CGA concentrations than wild Arabica. Da Silva Portela et al. [2022] demonstrated that coffee species is the primary determinant of chemical composition and sensory attributes, followed by roast level and brewing method. Deep roasting markedly reduces TG and CGA concentrations, whereas espresso extraction increases soluble solids and CAF relative to filter coffee. These patterns are consistent with the well-established coffee roasting chemistry. Laukaleja and Kruma [2019] described multiple chemical transformations during roasting, including polyphenol degradation (particularly CGAs), TG conversion to nicotinic acid, and Maillard reactions between proteins and sugars. Accordingly, light roasts retain higher levels of CGAs and antioxidant capacity but may exhibit greater acidity and bitterness, whereas dark roasts contain higher concentrations of caramelized and bitter compounds. Nur et al. [2023] also reported that medium-roasted Arabica often provides the best balance among acidity, body, and aroma and is associated with high consumer preference.

Despite these advances, routine identification of coffee species and blend composition in the marketplace still relies heavily on sensory evaluation by trained cuppers. Although sensory testing is intuitive and instrument-free, it is inherently subjective, difficult to quantify, and typically detects Robusta adulteration only when the Robusta content exceeds 35% [Cestari, 2021]. To improve objectivity, analytical chemistry approaches such as HPLC are commonly employed for CAF quantification [Klikarová and Česlová, 2022]. However, HPLC requires expensive instrumentation, skilled operators, and relatively long analysis times, resulting in high per-sample costs (∼ USD 100). Nuclear magnetic resonance (NMR) spectroscopy has also been used for coffee authentication. Lin et al. [2023] quantified 16-*O*-methylcafestol (16-OMC), a marker compound characteristic of Robusta but nearly absent in Arabica. Farag et al. [2022] used NMR-based metabolomics to profile nonvolatile constituents across roasting and brewing conditions. Although robust, NMR remains cost- and time-intensive, limiting its practicality for routine screening.

Spectroscopic techniques, including Fourier-transform infrared (FTIR) and near-infrared (NIR) spectroscopy coupled with chemometrics, have been proposed as faster and nondestructive alternatives. Briandet et al. [1996] quantified Arabica-to-Robusta ratios in instant coffee based on CAF and CGA compositional differences. Ribeiro et al. [2021] demonstrated accurate prediction of key compounds in green and roasted coffees using NIR combined with partial least squares (PLS) regression. Konieczka et al. [2020] applied NIR with PLS-DA and PLS models to discriminate pure coffees from samples adulterated with grains and estimate adulteration levels. Although NIR requires little or no reagents during measurement, outsourced UV/VIS/NIR analytical services can still be expensive, which may limit its practical cost advantage for routine sample analysis. Nevertheless, these spectroscopic techniques are often costly, may require complex preprocessing workflows, and are typically not portable, limiting their deployment for rapid on-site inspection.

Electrochemical sensing offers several advantages for food authentication, including rapid analysis, real-time operation, low cost, high sensitivity, and portability [Tyszczuk-Rotko & Bęczkowska, 2015]. Beyond conventional electroanalytical applications, portable, miniaturized electrochemical platforms, particularly those based on disposable three-electrode chips and nanostructured nanogold-modified electrodes, can provide reliable, user-friendly detection of a wide range of biomolecular targets. For example, portable voltammetric chips have been successfully applied for rapid bacterial detection, whereas nanostructured electrochemical biosensors have enabled the sensitive determination of peptide aggregation, autoantibodies, and protein biomarkers in biological samples [Fan et al., 2020; Wang et al., 2022; Shen et al., 2025]. These studies highlight the practical feasibility of compact electrochemical systems for rapid and low-sample-consumption analysis. Such developments considerably expand portable electrochemical sensing to coffee authentication and blend analysis, where simple operation, short analysis time, and field-deployability are essential.

With the expansion of computational capabilities, machine learning (ML) has become a central component of artificial intelligence (AI), enabling data-driven modeling across diverse scientific domains [Jordan & Mitchell, 2015]. In electrochemical sensing, Zhao et al. [2021] employed ML to interpret electrochemical responses for glucose and insulin detection and Alharbi et al. [2015] applied ML to calibrate Raman spectra for quantifying CAF and related compounds. In coffee authentication, Tsai et al. [2023] developed an artificial neural network (ANN) using mass spectrometry features to distinguish civet coffee from regular Arabica and non-Arabica samples. [Briandet et al., 1996] combined attenuated total reflection-Fourier transform mid-infrared spectroscopy (ATR-FTIR) with principal component analysis (PCA) and partial least squares-discriminant analysis (PLS-DA) to achieve 100% classification accuracy between Arabica and Robusta coffees. Meanwhile, Gomes et al. [2022] used ensemble ML models (random forest (RF) and extreme gradient boosting (XGBoost)) on multispectral imaging data to classify Arabica and commercial green coffees. Further, ML can enhance the robustness of portable electrochemical sensors by compensating for signal variations arising from sensor batch differences and operational conditions, such as waiting time [Gu et al., 2023]. ANN-assisted calibration strategies are particularly attractive for coffee analysis, where rapid and reliable field measurements are essential. Recent developments in other sensing domains have similarly demonstrated that ML can improve the interpretation and robustness of complex multimodal measurements. For example, Ji et al. [2026] combined LIBS and laser-induced acoustic signals with feature-selection-based random forest modeling for robust aerosol identification, while Ye et al. [2026] introduced a physics-aware acoustic–optical fusion strategy for intelligent monitoring of industrial aerosols. These advances further illustrate the broader trend toward integrating physical sensing with data-driven models to improve classification robustness under complex measurement conditions.

More recent studies have further demonstrated the potential of compact sensing and machine-learning approaches for coffee authentication. Sagita et al. [2025] combined an affordable multi-channel spectral sensor with ML for rapid discrimination of Arabica and Robusta species and geographical origins, achieving 100% species-classification accuracy. Suhandy et al. [2025] employed portable LED-based fluorescence spectroscopy with chemometric models for rapid authentication and quantitative assessment of adulteration in ground-roasted Arabica coffee. More recently, Malavi et al. [2026] integrated NIR hyperspectral imaging with chemometrics, feature selection, and ML for both detection and quantification of Robusta adulteration in ground and instant Arabica coffee, achieving external-test balanced accuracies exceeding 98% with optimized classifiers. These studies demonstrate the growing importance of combining portable or non-destructive sensing with data-driven modeling for rapid coffee authentication. However, most recent approaches remain spectroscopy-based, motivating the development of complementary electrochemical strategies that provide chemically interpretable compositional descriptors for ML analysis.

Building on these advances, we integrate ML with electrochemical sensing to establish a portable, real-time, and cost-effective framework for coffee species classification and blend quantification. Electrochemical measurements of CAF, CGAs, TG, and reducing sugars (RS), combined with roast-level information, were used to train ML models for the rapid identification of coffee varieties and estimation of Arabica and Robusta blending ratios. This approach addresses the primary limitations of conventional chromatographic and spectroscopic methods, such as high cost, lengthy analysis time, and limited field-deployability, while highlighting the potential of AI-assisted electrochemical sensing for food authentication and quality control.

## 2 Materials and methods

Fig. 1 summarizes the overall workflow. After determining the roast level, coffee powders from Arabica, Robusta, and blended samples were analyzed using a portable electrochemical sensing device. Electrochemical responses corresponding to CAF, CGAs, TG, and RS were extracted as quantitative descriptors and used as input variables for ML models. XGBoost was employed for coffee-species classification (Arabica, Robusta, or blend), whereas an ANN model was used to predict Arabica–Robusta blending ratios.

**Fig. 1:**
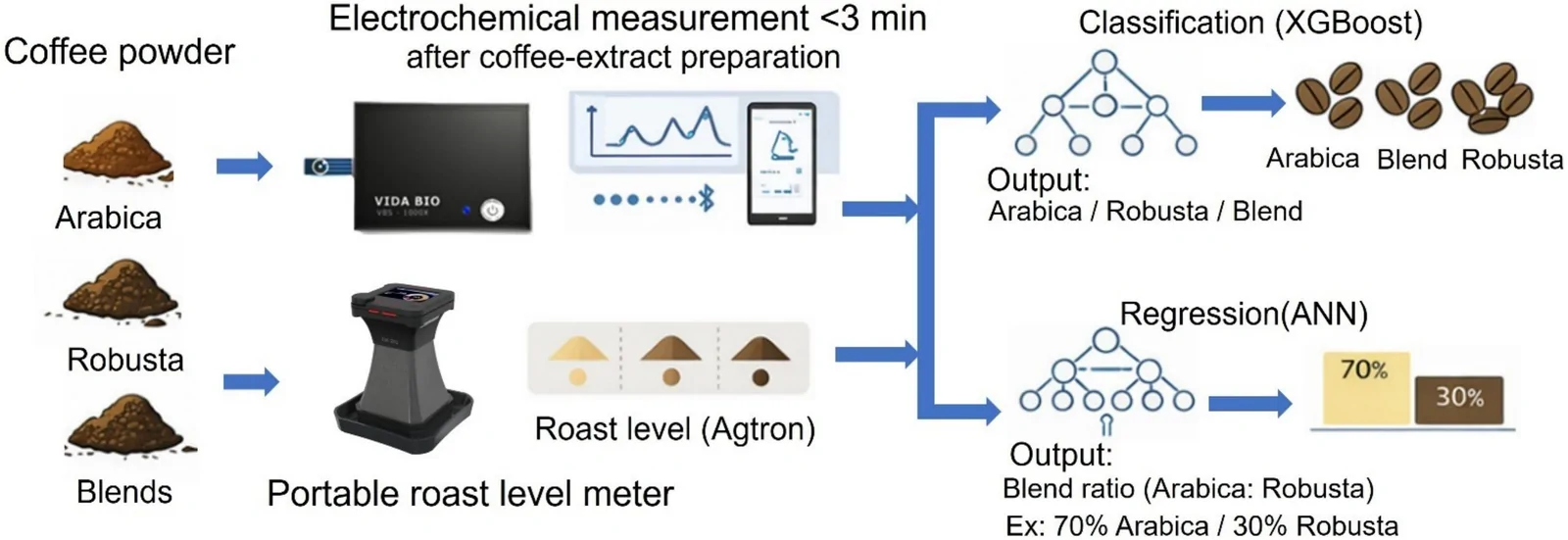
Workflow of the portable electrochemical sensing–ML platform for coffee authentication and blend-ratio prediction.

### 2.1 Experimental setup and materials

#### 2.1.1 Apparatus

Fig. S1 shows the main instruments and materials used in this study, including (1) a coffee roast level meter (Lighttells Co., Ltd., Taiwan); (2) a portable electrochemical analyzer (VIDA Bio Tech. Co., Ltd., Taiwan); (3) coffee samples (Lighttells Co., Ltd., Taiwan); and (4) disposable screen-printed sensor chips (VIDA Bio Tech. Co., Ltd., Taiwan) with a three-electrode configuration. Carbon electrodes were used to measure CAF, CGAs, and TG, an Au electrode was used for sugar-related measurements, and Ag/AgCl served as the reference electrode (Fig. S1B). Figs. S1A and S1B show the roast level meter and portable analyzer, respectively.

Electrolytes were prepared as follows:

• Electrolyte A for CAF and CGA detection (0.05-M H_2_SO_4_; 900 μL)
• Electrolyte B for TG detection (0.003-M NH_4_OH; 700 μL)
• Electrolyte C for RS detection (0.1-M NH_4_OH, 400 μL, mixed with 0.5-M CuSO_4_, 500 μL).

The experimental setup, including all instruments and materials, is shown in Fig. S1.

#### 2.1.2 Coffee samples

Robusta beans from Vietnam, India, and Brazil were roasted to three levels (light, medium, and dark), with two replicates per condition, yielding 18 samples in total. Arabica samples comprised 11 varieties from Ethiopia, Kenya, and Colombia (Baby Geisha, Sidamo G2, Wulaga, Yirgacheffe Perfume Lemon, Yirgacheffe Star, Dobby Rabbit, Pink Elephant, Grey Elephant, G2 Ethiopia, Kenya Golden Elephant, and Colombia Huila), each roasted to the same three levels, producing a total of 66 samples. Roast level was determined by grinding the beans and measuring Agtron values using the roast level meter.

The coffee origins and varieties were selected to provide both commercial relevance and within-species compositional diversity. Robusta samples from Vietnam, India, and Brazil were included to represent geographically distinct production regions, whereas the Arabica set incorporated multiple varieties from Ethiopia, Kenya, and Colombia, covering both East African and Latin American origins. The relatively larger number of Arabica varieties was intended to capture broader within-species variability rather than characterize a single cultivar or origin. In combination with the three controlled roast levels, this design allowed the dataset to incorporate variability associated with origin, variety, and roasting while retaining consistent sample preparation and analytical conditions. The selected coffees were not intended to represent the complete global diversity of either species, and additional origins and harvest seasons should be incorporated in future validation studies.

Blended samples were formulated based on measured roast level to minimize roast-related bias. Arabica and Robusta beans were first roasted separately to the designated roast levels. The roasted beans were then weighed and combined on a mass basis at Arabica/Robusta ratios of 95:5, 90:10, 80:20, 70:30, 50:50, and 30:70 before grinding and brewing. Thus, the reported blend ratios represent the mass fractions of roasted beans, rather than green-bean mass or brewed-liquid volume. This procedure minimizes potential bias arising from species-dependent moisture loss during roasting. Representative blend pairs included light-roast Ethiopia G2 with Brazil Robusta, medium-roast Yirgacheffe Perfume Lemon with India Robusta, and dark-roast Sidamo G2 with Vietnam Robusta. This matched-roast design was intentionally used to minimize roast-level differences as a confounding factor during model development.

#### 2.1.3 Coffee brewing procedure

To minimize variability in preparation, coffee brewing followed the Specialty Coffee Association of America protocol. Whole beans were ground for 5 min, sieved through a 40-mesh filter, and transferred into a cup. Boiling water was added at a ratio of 1-g coffee to 18.18-mL water. The slurry was stirred for 30 s and allowed to stand for 5 min. The extract was then filtered and used for subsequent electrochemical measurements.

### 2.2 Electrochemical measurements

The electrochemical sensing strategy was designed based on established reaction pathways (Figs. S1C–S1F), including anodic oxidation of CAF (Fig. S1C) and CGAs (Fig. S1D), alkaline conversion of TG to nicotinic acid before detection (Fig. S1E), and Cu^2+^/Cu₂O redox chemistry for RS quantification under alkaline conditions (Fig. S1F). Our previous study also demonstrated good agreement between electrochemical and HPLC measurements of CAF and CGA [Gu et al., 2023], providing complementary analytical support for the present platform. In the current study, additional independent laboratory comparisons for CAF, CGA, TG, and sugar-related measurements are provided in Table S8.

#### 2.2.1 Detection of CAF and CGAs

A 100-μL aliquot of brewed coffee was mixed with 900 μL of electrolyte A to provide an acidic medium for electrochemical measurement. A 200-μL droplet of the mixture was applied to the working electrode, fully covering the active area without trapping air bubbles. Linear sweep voltammetry (LSV) and differential pulse voltammetry (DPV) were employed.

(1) LSV parameters: potential range of 50–800 mV; scan rate of 50 mV s^−1^; initial potential of 50 mV.
(2) DPV parameters: potential range of 0–1600 mV; initial potential of 0 V; pulse height of 5 mV; pulse width of 5 ms; step height of 5 mV; step time of 300 ms.

#### 2.2.2 Detection of TG

A 300-μL coffee sample was mixed with 700 μL of electrolyte B (0.003 M NH4OH). Under alkaline conditions, TG is converted to nicotinic acid (Fig. S1E). A 200-μL droplet was applied to the electrode and measured by DPV with the following parameters: initial potential of 1000 mV; final potential of 1800 mV; pulse height of 5 mV; pulse width of 5 ms; step height of 5 mV; step time of 300 ms.

#### 2.2.3 Detection of RS

For sugar-related measurements, 100-μL coffee extract was combined with 900 μL of electrolyte C (0.1-M NH_4_OH containing 0.5-M CuSO_4_). Under alkaline conditions, RS reduces Cu^2+^ to Cu_2_O (Fig. S1F). A 200-μL droplet of the mixture was applied to the electrode and analyzed by DPV. The measurement parameters were as follows: initial potential of −600 mV; final potential of 200 mV; pulse height of 100 mV; pulse width of 100 ms; step height of 20 mV; and step time of 200 ms.

#### 2.2.4 Analytical validation of the electrochemical measurements

The analytical performance of the portable electrochemical platform was validated for caffeine (CAF), chlorogenic acids (CGA), trigonelline (TG), and reducing sugars (RS) under the measurement conditions described in Sections 2.2.1–2.2.3. All validation experiments were conducted at 25 °C. The validation parameters included calibration linearity and working range, limits of detection and quantification, accuracy by spike recovery, precision/reproducibility across disposable sensor chips and production batches, matrix effects, and comparison with independent external laboratory measurements using standardized or laboratory-developed HPLC procedures, as appropriate for each analyte.

Calibration standards were prepared over concentration ranges of 20–500 mg dL^−1^ for CAF and CGA, 10–100 mg dL^−1^ for TG, and 65–1250 mg dL^−1^ for RS. Each calibration level was measured using 3 independent replicates and 3 independent disposable chips were used. The analytical responses used for calibration were CAF_I_2_, CGA_I_1_, TG_I, and RS_I, as defined in Fig. S3. Calibration equations were obtained by least-squares linear regression:

(1) y=aC+b

where C is the analyte concentration, y is the electrochemical response, a is the calibration slope, and b is the intercept. Linearity was evaluated using the coefficient of determination (R^2^) and residual distribution. The complete calibration characteristics are reported in Table S5, and the corresponding calibration plots are shown in Fig. S7. The limits of detection (LOD) and quantification (LOQ) were calculated as

(2) $$\mathrm{LOD}=3.3\sigma_{c}$$

(3) $$\mathrm{LOQ}=10\sigma_{c}$$

where $\sigma_{c}$ is the concentration-domain standard deviation used for LOD and LOQ estimation. The experimentally determined LOD and LOQ values were 6.60 and 20.00 mg dL^−1^ for CAF, 5.02 and 15.21 mg dL^−1^ for CGA, 1.26 and 3.83 mg dL^−1^ for TG, and 19.27 and 58.40 mg dL^−1^ for RS, respectively. Where applicable, quantification performance near the calculated LOQ was verified by replicate measurements with acceptable precision and recovery. Method accuracy was evaluated by standard-addition experiments performed directly in representative coffee matrices. Accuracy was evaluated by standard addition at two representative concentration levels in Arabica and Robusta coffee matrices. Each spiked and unspiked sample was analyzed in **3** independent measurements. Recovery was calculated as:

(4) $$\mathrm{Recovery}\left(\%\right)=\frac{C_{\mathrm{spiked}}-C_{\mathrm{unspiked}}}{C_{\mathrm{added}}}\times 100$$

where C_spiked_ and C_unspiked_ are the measured concentrations in the fortified and original samples, respectively, and C_added_ is the added analyte concentration. A recovery range of **80–120%** was considered acceptable. Detailed analyte-, matrix-, and spike-level recovery results are provided in Table S6A. Precision was assessed at **a representative** concentration for each analyte. Repeatability was evaluated under the same operating conditions, including the same analyst, day, instrument, and sensor-chip batch. Because the disposable chips were intended for single use, replicate measurements were performed using independent chips from the same production batch unless otherwise specified. Inter-batch reproducibility was evaluated using 15 independently selected disposable chips from three independently manufactured batches, with five chips evaluated per batch. Precision was expressed as the relative standard deviation:

(5) $$\mathrm{RSD}\left(\%\right)=\frac{\mathrm{SD}}{\bar{x}}\times 100$$

where $\bar{x}$ and SD are the mean and standard deviation of the measured concentration or electrochemical response. The analyte-specific precision and inter-batch reproducibility results obtained using independent disposable chips are summarized in Table S7.

Matrix effects were assessed by comparing the slopes of calibration curves prepared in standard solution with those obtained using matrix-matched calibration or standard addition in representative Arabica and Robusta coffee extracts. Where applicable, different roast levels were included to determine whether roasting-derived constituents affected the electrochemical response. The slope ratio and signal suppression or enhancement were calculated as:

(6) $$\mathrm{Slope ratio}\;\left(\%\right)=\frac{S_{\mathrm{matrix}}}{S_{\mathrm{Standard}}}\times 100$$

(7) $$\mathrm{Matrix effect}\;\left(\%\right)=\left(\frac{S_{\mathrm{matrix}}}{S_{\mathrm{Standard}}}-1\right)\times 100$$

To further assess the analytical plausibility of the electrochemical measurements, selected coffee samples were independently analyzed by JITTEC Service Corporation (Taiwan). The external analytical procedures differed according to the target constituent. Caffeine was determined according to CNS 9432 (N6174), Method of test for caffeine content in foods. Sugar analysis was performed according to CNS 12634 (2006), Method of test for fruit and vegetable juice products – Determination of sugars by HPLC, which determines fructose, glucose, sucrose, maltose, and lactose; total-sugar concentrations were additionally reported by the laboratory.

For chlorogenic acids (CGA) and trigonelline (TG), no official TFDA method specifically applicable to coffee samples was used in the present study. These analytes were therefore determined by JITTEC using its laboratory-developed HPLC procedures. The detailed chromatographic conditions of these in-house procedures, including the column, mobile phase, detector configuration, and internal calibration protocol, were not provided in the issued analytical results and are therefore not reproduced here.

Accordingly, the JITTEC measurements were used as independent external laboratory comparators rather than being uniformly described as fully reproduced reference methods. For CAF and sugars, standardized CNS methods were available, whereas the CGA and TG comparisons were based on independent laboratory HPLC measurements. In addition, because the electrochemical RS assay reflects a reducing-sugar-related response whereas CNS 12634 reports individual sugars and total sugars, direct equivalence between the two measurands was not assumed. The sugar comparison was therefore interpreted as supporting consistency in sugar-related analytical trends rather than strict method equivalence. Further details of the external laboratory analyses and individual comparison results are provided in Section S2 and Table S8 of the Supplementary Information.

### 2.3 ML model development

Electrochemical and roasting data were analyzed using ML models to classify coffee species and predict blend ratios. The input feature set included CAF, CGAs, TG, RS, and roast level, capturing both compositional and roasting-related characteristics of the coffee samples.

The dataset comprised 138 samples: 66 Arabica, 18 Robusta, and 54 blended coffees (Table 1). The original random split was retained as a within-distribution benchmark. To provide a more conservative assessment of model generalization, additional group-aware validation strategies were implemented separately for species classification and blend-ratio regression, as described in Sections 2.3.3 and 2.3.4. Although the dataset is moderately imbalanced due to the smaller number of Robusta samples, all classes were retained to reflect realistic market composition.

**Table 1:** Sample dataset of coffee.

Type	Country/ Varieties		Roast classification (Agtron)					Number of data entries	
			**Light**		**Medium**		**Dark**		
Robusta	Vietnam		128.1		103		64.9	18	
	India		131.8		99		70		
	Brazil		126.5		87.9		61.8		
Arabica	Ethiopia	Baby Geisha	69.1		59.3		50.2	66	
		Wulaga	82.4		80.3		51.1		
		Pink Elephant	94		70		60		
		Yirgacheffe Perfume Lemon	91		65.9		50.4		
		Yirgacheffe Star	85.9		68.7		50.1		
		Sidamo G2	75.4		65.8		48.9		
		Grey Elephant	93		92		59.8		
		Dobby Rabbit	97.1		78		55.8		
		G2 Ethiopia	124.6		80.2		36.9		
	Kenya	Golden Elephant	82		63		43		
	Colombia	Huila	114.7		60.8		29.8		

Blend	Blending type		**Blending ratio**						
			95:5	90:10	80:20	70:30	50:50	30:70	
			**Roast level (Agtron)**						**Number of data entries**
	Ethiopia G2+ Brazil		124.7	124.79	124.98	125.17	125.55	125.93	54
	Sidamo G2+ Vietnam		91.4	91.8	92.6	93.4	95	96.6	
	Yirgacheffe Perfume Lemon + India		65.76	65.71	65.62	65.53	65.35	65.17	

Two classification algorithms, RF and XGBoost, were evaluated for coffee species identification. Model hyperparameters were optimized using cross-validation within the training dataset. Performance was assessed using classification accuracy and a confusion matrix.

To predict Arabica–Robusta blend ratios quantitatively, an ANN regression model was developed using the same feature set. Model performance was evaluated using the coefficient of determination (R^2^), root mean square error (RMSE), and mean absolute error (MAE). Hyperparameter optimization and architecture selection for the ANN regression model are detailed in Section S1 (Supplementary Information).

#### 2.3.1 Model overview

The model frameworks are summarized in Figs. S1G–S1I and include RF and XGBoost for classification and the ANN model for blend-ratio regression. Two tasks were defined: (i) three-class classification (Arabica/Robusta/blend) using RF and XGBoost, and (ii) continuous regression of blend ratios using the ANN model.

All ML analyses were implemented in Python. RF and XGBoost classification were implemented using scikit-learn and XGBoost, respectively, whereas the final blend-ratio regression framework was implemented in PyTorch. Where applicable, fixed random seeds were used to improve computational reproducibility. Table S1 summarizes the model settings and search ranges used in the present study. The original random train/test split and conventional 10-fold cross-validation were retained as within-distribution benchmarks (Fig. S2A), whereas stricter group-aware validation was performed separately for origin-level classification and blend-ratio regression, as described in Sections 2.3.3 and 2.3.4.

Input variables comprised five electrochemical and physical descriptors: CAF, CGA, TG, and RS concentrations together with coffee roast level. The output corresponded to either a categorical class label for classification or a continuous Arabica blend fraction for regression.

For coffee-species classification, RF was implemented with 300 trees and out-of-bag evaluation enabled (Table S1). XGBoost employed 400 boosting rounds with a learning rate of 0.05, a maximum tree depth of 4, and subsampling/feature-sampling ratios of 0.8. The classification models were evaluated using overall accuracy, balanced accuracy, class-specific precision, recall, and F1-score, macro-averaged precision, recall, and F1-score, and confusion matrices.

For blend-ratio regression, the original random-split ANN result was retained as a within-distribution benchmark, while the final robustness assessment used a nested group-aware ANN ensemble framework. Rather than relying on a single fixed ANN architecture, a candidate pool containing 16 ANN base architectures evaluated under three random seeds was considered within each outer training fold, yielding 48 candidate models per fold. Candidate hidden-layer configurations included 500, 500–128, 512–256, 384–192, and 256–128 neurons, together with regularized, noise-augmented, balanced, blend-focused, and multi-task variants. ReLU6 was used as the activation function, Huber loss as the primary regression loss, and Adam as the optimizer.

Within each outer training fold, model selection was performed using a dual-inner consensus strategy combining pseudo-leave-one-blend-pair-out validation and repeated stratified inner validation. Four unique ANN architectures showing the most stable performance across the two inner-validation schemes were selected and combined using fixed rank-based ensemble weights of 0.35, 0.25, 0.25, and 0.15. The detailed ANN search space, optimization procedure, and ensemble construction are described in Section S1 and Table S1 of the Supplementary Information.

All preprocessing and model-selection procedures were restricted to the corresponding training data. Min–Max scaling parameters were estimated exclusively from the training fold and then applied unchanged to the associated validation and held-out test samples. The outer held-out samples were not used for scaling, architecture selection, hyperparameter tuning, epoch selection, ensemble construction, calibration, or post-processing.

Regression performance was quantified using the coefficient of determination R^2^, root mean square error (RMSE), and mean absolute error (MAE). In addition, a prediction was considered practically acceptable when the absolute deviation from the reference blend fraction did not exceed ±0.10. All reported regression metrics were calculated from the original continuous ANN outputs; no reference normalization, post-hoc calibration, or nearest-label snapping was applied.

#### 2.3.2 Dataset preprocessing and feature extraction

Electrochemical profiles of CAF, CGAs, TG, and RS were processed to extract features for ML. Roast level was included as an additional descriptor. For the original random-split evaluation, the dataset was first divided into training (72.5%) and test (27.5%) sets. Min–Max scaling parameters were then estimated exclusively from the training set and applied unchanged to the test set. For cross-validation and group-aware evaluations, scaling parameters were independently fitted within each corresponding training fold and subsequently applied to the validation or held-out test data. No information from the held-out samples was used during preprocessing:

(8) $$x_{\mathrm{normalized}}=\frac{x-x_{\min}}{x_{\max}-x_{\min}}$$

Voltammetric curves were transformed into compact, physically interpretable features (Fig. S3).

For CAF, two characteristic DPV peak regions were quantified by recording peak current and potential (CAF_I_1_, CAF_V_1_; CAF_I_2_, CAF_V_2_) and by computing baseline-corrected integrated peak areas (CAF_A_1_ and CAF_A_2_) within predefined potential windows (Fig. S3A). Analogous descriptors were extracted for CGAs from LSV and DPV profiles, including (CGA_I_1_, CGA_V_1_, CGA_A_1_) and (CGA_I_2_, CGA_V_2_, CGA_A_2_) after baseline correction (Fig. S3B). TG features were defined as TG_I, TG_V, and TG_A using the same baseline-corrected peak integration procedure (Fig. S3C). Sugar-related signals were represented by Sugar_I and Sugar_V (Fig. S3D).

These features served as model inputs to improve robustness across different coffee origins and roast levels. They constitute physically interpretable electrochemical descriptors for ML models. Blend ratios were assigned according to experimentally prepared mixtures (0, 0.3, 0.5, 0.7, 0.8, 0.9, 0.95, and 1.0) to reflect realistic commercial blending scenarios.

#### 2.3.3 Model training and validation

RF and XGBoost were trained to classify Arabica, Robusta, and blended samples. Feature importance analyses quantified the contributions of CAF, CGAs, TG, sugar-related features, and roast level.

To assess robustness and mitigate overfitting, 10-fold cross-validation (k = 10) was performed (Fig. S2A). The training dataset was partitioned into 10 folds; in each iteration, one fold was held out for validation, and the remaining 9 were used for training. This procedure was repeated until each fold had served as the validation set once, yielding fold-level metrics *E*_*1*_ ∼ *E*_*10*_. Overall performance was reported as the mean across folds:

(9) $$E=\frac{1}{10}\sum_{i=1}^{10}E_{i}$$

For classification analysis, no explicit class-weighting or resampling strategy was applied to handle the moderate class imbalance in the dataset. The classification models were evaluated using overall accuracy, balanced accuracy, class-specific precision, recall, and F1-score, macro-averaged precision, recall, and F1-score, and confusion matrices.

To further evaluate whether coffee-species discrimination was retained for origins that were not represented during model training, an additional leave-one-origin-out (LOOO) analysis was conducted using the pure Arabica and Robusta samples. In each LOOO iteration, all samples associated with one coffee origin were withheld from model development and used exclusively for testing, whereas the remaining origins were used for model training. Because blended samples contain two coffee sources and therefore cannot be assigned unambiguously to a single-origin holdout group, the LOOO analysis was used specifically to evaluate pure-bean Arabica-versus-Robusta discrimination. Accordingly, this origin-level binary classification task was treated separately from the original three-class Arabica/Robusta/blend classification.

For all held-out evaluations, data preprocessing was performed within each training fold to avoid information leakage. Min–Max scaling parameters were estimated exclusively from the corresponding training data and then applied unchanged to the validation and held-out test samples. No scaling parameters, feature-selection decisions, or model-selection information were derived from the held-out samples.

For blend-ratio prediction, the original random train/test split was retained as a within-distribution benchmark and was evaluated using the coefficient of determination R^2^, root mean square error (RMSE), and mean absolute error (MAE). A prediction was additionally considered practically acceptable when the absolute deviation from the reference blend fraction was within ±0.10.

Baseline regression models, including RF regression and multiple linear regression (MLR), were implemented for comparative benchmarking. However, because random splitting may distribute samples derived from the same Arabica–Robusta pairing across both the training and test subsets, a stricter group-aware validation procedure was additionally implemented for the ANN regression model, as described in Section 2.3.4.

#### 2.3.4 Nested group-aware validation for blend-ratio regression

To address the potential optimistic bias associated with random sample splitting, a nested group-aware validation framework was implemented for Arabica–Robusta blend-ratio regression. The blended dataset comprised three independent Arabica–Robusta pairings: Ethiopia G2 + Brazil Robusta, Sidamo G2 + Vietnam Robusta, and Yirgacheffe Perfume Lemon + India Robusta. Each pairing contained 18 blended samples spanning the experimentally prepared blend ratios.

The outer validation loop used a leave-one-blend-pair-out (LOBPO) design. In each outer fold, all 18 samples belonging to one complete Arabica–Robusta pairing were withheld from model development and used only for final testing. The remaining 120 samples were used for preprocessing, model selection, and model fitting. Thus, different blend ratios derived from the same coffee pair were never divided between training and testing, preventing the model from benefiting from prior exposure to the same blend formulation.

All five input variables—roast level, CAF, CGA, TG, and RS—were retained throughout the group-aware analysis. Min–Max normalization was fitted exclusively to the outer-training data and subsequently applied to the corresponding inner-validation and outer-test samples.

Within each outer-training fold, ANN model selection was performed using a dual-inner consensus strategy. Two complementary inner-validation procedures were used: (i) pseudo-LOBPO validation, in which one complete blend pair within the outer-training data was withheld as an inner validation group, and (ii) repeated stratified inner validation using three independent splits with a validation fraction of 0.25. Candidate ANN models were ranked according to their performance across both inner-validation schemes so that model selection did not depend on a single partitioning strategy.

The ANN candidate pool consisted of 16 base architectures evaluated using three random seeds, yielding 48 candidate models per outer fold. Candidate hidden-layer configurations included 500, 500–128, 512–256, 384–192, and 256–128 neurons, together with regularized, noise-augmented, balanced, blend-focused, and multi-task variants. ReLU6 was used as the activation function, Huber loss as the primary regression loss, and Adam as the optimizer. Dropout rates ranged from 0.05 to 0.10, learning rates from $1\times 10^{-4}$ to $2\times 10^{-4}$, and weight decay from 0 to $1\times 10^{-5}$. Batch sizes of 32 or 35 were evaluated.

Training within the inner loop was limited to a maximum of 1200 epochs, with early stopping patience of 90 epochs. The learning rate was reduced on plateau using a patience of 30 epochs, a reduction factor of 0.5, and a minimum learning rate of $1\times 10^{-6}$.

For each outer fold, four unique ANN architectures showing the most stable consensus performance across the inner-validation procedures were selected to form the final ensemble. Fixed rank-based ensemble weights of 0.35, 0.25, 0.25, and 0.15 were applied to the four selected members. Each selected member was refitted using random seeds 7, 37, and 73, and the final training duration was determined as 1.30 times the inner-selected epoch, constrained to 120–1400 epochs.

The final ensemble prediction was calculated from the weighted average of the continuous outputs of the selected ANN members. No reference normalization, post-hoc calibration, or nearest-label snapping was applied. Multi-task ANN variants included an auxiliary blend-ratio classification head during training; however, the reported prediction remained the continuous regression output.

To quantify the stability of the ANN refitting process, repeated grouped evaluations were also conducted using the same three unique blend-pair holdout partitions. These repeated runs were used to estimate variability arising from stochastic ANN initialization and refitting and were not interpreted as additional independent group partitions.

Group-aware regression performance was evaluated using R^2^, RMSE, MAE, and the fraction of predictions within ±0.10 of the reference blend fraction. Both pooled LOBPO performance across the three outer folds and the results for each individual held-out blend pair were reported. The complete validation workflow and ANN search settings are summarized in Section S1 and Table S1 of the Supplementary Information. The nested group-aware ANN ensemble validation workflow is summarized in Fig. S2B.

## 3 Results and discussion

### 3.1 Electrochemical profiling of key coffee constituents and roast-dependent trends

Electrochemical signatures of CAF, CGAs, TG, and RS were reproducibly obtained using the portable three-electrode platform. Characteristic responses were consistent across coffee samples (Fig. S3), enabling multi-constituent analysis with microliter-scale operation. Following preparation of the coffee extract, the electrochemical measurement step could be completed within 3 min. This measurement time does not include grinding, brewing, standing, or filtration. CAF and CGA exhibited anodic features in the low-potential region (≈200–300 mV), consistent with the previously reported oxidation behavior of CAF and phenolic CGA structures. TG displayed a prominent oxidation peak at a high potential (∼1500 mV), attributable to the alkaline conversion of TG to electroactive nicotinic acid-related species before detection. RS produced a characteristic response in the negative potential region (≈ −250 mV), consistent with Cu^2+^/Cu₂O redox chemistry under alkaline conditions (Figs. S1C–S1F). Peak-derived descriptors were extracted as described in Section 2.3.2 and are illustrated in Fig. S3.

### 3.2 Analytical performance and validation of the portable electrochemical platform

The analytical performance of the portable electrochemical platform was evaluated for caffeine (CAF), chlorogenic acids (CGA), trigonelline (TG), and reducing sugars (RS) at 25 °C. The validation included calibration linearity and working range, limits of detection and quantification, spike recovery, precision across disposable sensor chips and production batches, matrix effects, and comparison with independent laboratory measurements. Detailed validation results are provided in Fig. S7 and Tables S5–S8 of the Supplementary Information.

Linear calibration relationships were obtained over concentration ranges of 20–500 mg dL^−1^ for CAF and CGA, 10–100 mg dL^−1^ for TG, and 65–1250 mg dL^−1^ for RS. The corresponding calibration equations were y = 16.70C + 171.80 for CAF, y = 3.50C + 4.95 for CGA, y = 1.19C + 9.18 for TG, and y = 0.0904C + 15.21 for RS, where C is the analyte concentration and y is the extracted electrochemical response. The limits of detection and quantification were calculated as $3.3\sigma_{c}$ and $10\sigma_{c}$, respectively, where $\sigma_{c}$ is the concentration-domain standard deviation used for LOD and LOQ estimation. The resulting LOD/LOQ values were 6.60/20.00 mg dL^−1^ for CAF, 5.02/15.21 mg dL^−1^ for CGA, 1.26/3.83 mg dL^−1^ for TG, and 19.27/58.40 mg dL^−1^ for RS. In all cases, the LOQ was at or below the lower limit of the corresponding working range, supporting quantitative determination over the selected concentration intervals.

Because analytical validation of TG and RS was of particular importance in the present study, recovery was further assessed by standard addition in representative Arabica and Robusta coffee matrices. TG recoveries ranged from 97.2% to 110.3%, with RSD values of 1.35–3.59%, whereas RS recoveries ranged from 92.9% to 113.9%, with RSD values of 3.65–4.57% (Table S6A). These values were within the predefined acceptance range of 80–120%, indicating acceptable accuracy for TG and RS determination in the tested coffee matrices.

Precision and sensor reproducibility were evaluated using 15 independently selected disposable chips from three production batches, with five chips evaluated per batch. The resulting RSD values were 6.23% for CAF, 6.76% for CGA, 6.84% for TG, and 9.17% for RS (Table S7). These results indicate that the electrochemical measurements remained reasonably consistent despite variation among independently fabricated disposable sensors and production batches.

Matrix effects were evaluated by comparing matrix-matched calibration slopes with those obtained in standard solutions. Signal suppression or enhancement ranged from −0.7% to +0.3% for CAF, −2.8% to +6.0% for CGA, −0.7% to +1.7% for TG, and − 1.3% to +1.2% for RS (Table S6B). The corresponding slope ratios remained close to 100%, indicating limited matrix-induced alteration of the electrochemical responses in the tested Arabica and Robusta coffee extracts.

The electrochemical measurements were additionally compared with independent laboratory analyses performed by JITTEC Service Corporation (Taiwan). CAF was determined according to the standardized CNS 9432 method, whereas sugars were analyzed according to CNS 12634 (2006), an HPLC-based standardized method for individual sugar determination. CGA and TG were analyzed using JITTEC laboratory-developed HPLC procedures because no official TFDA method specifically applicable to coffee samples was used for these analytes. Detailed chromatographic conditions for the laboratory-developed procedures were not available to the authors; therefore, these results are treated as independent laboratory comparators rather than fully reproduced standardized reference methods. For RS, the external analysis reports individual and total sugars, whereas the electrochemical method reflects a reducing-sugar-related response; consequently, the comparison was interpreted as supportive evidence of analytical consistency rather than strict method equivalence. The individual comparison results are summarized in Table S8 and further described in Section S2 of the Supplementary Information. Overall, the calibration, recovery, precision, matrix-effect, and external-comparison results support the suitability of the portable electrochemical platform for generating quantitative chemical descriptors for subsequent ML analysis.

### 3.3 Roast-dependent compositional changes in representative coffee samples

To evaluate roasting-induced compositional changes, representative coffee samples were analyzed across four roast stages: green bean, light, medium, and dark. Fig. 2 summarizes the electrochemically quantified CGA, RS, TG, and CAF concentrations for three Arabica origins (G2 Ethiopia, Golden Elephant, and Huila Colombia) and one Robusta origin (Vietnam).

**Fig. 2:**
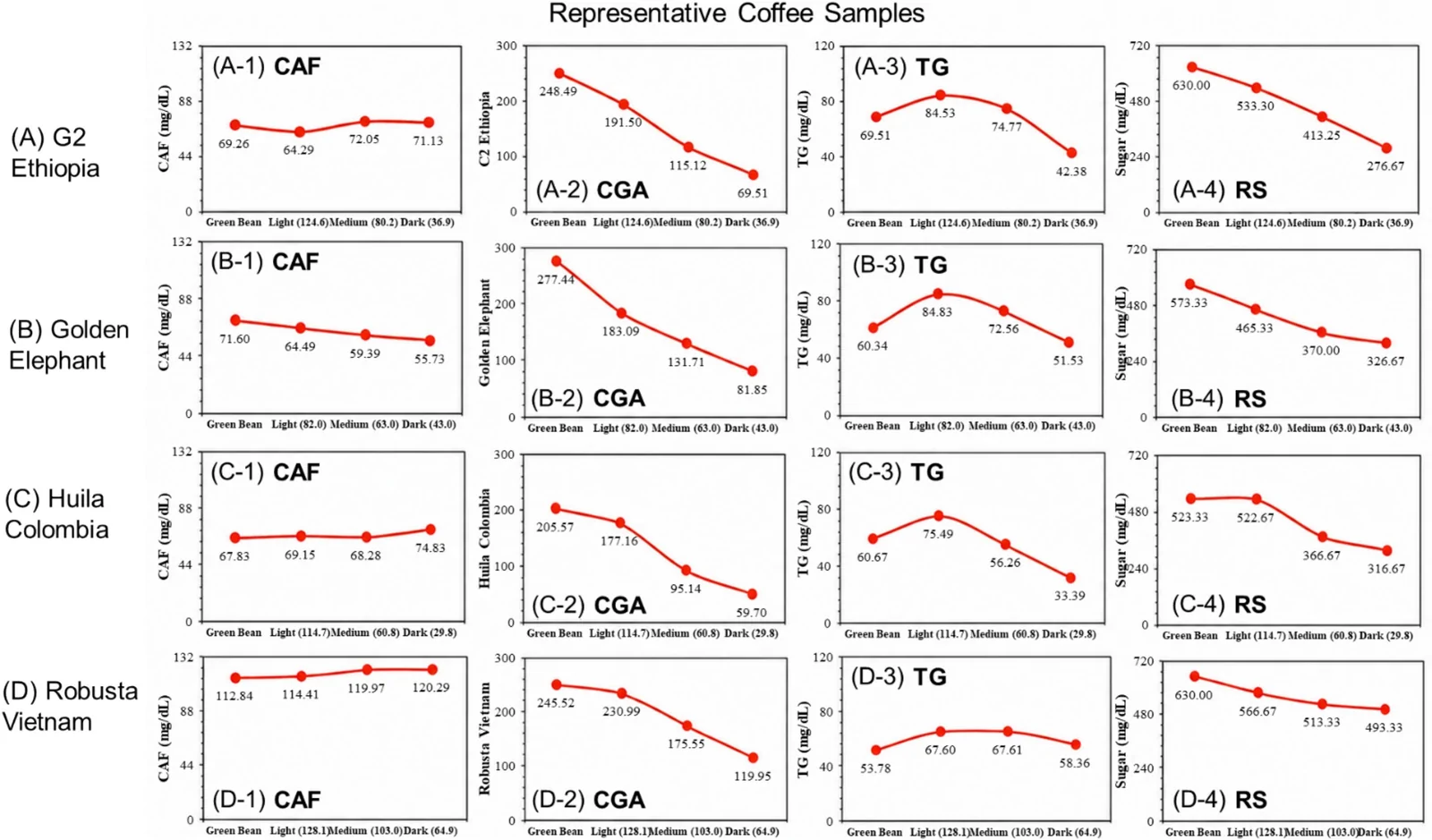
Roast-stage evolution of key coffee constituents in representative Arabica and Robusta samples. Representative Arabica coffees (G2 Ethiopia, Golden Elephant, and Huila Colombia) and a Robusta coffee (Vietnam) were analyzed across four roast levels (green bean, light, medium, and dark) using the portable electrochemical sensing platform. Panels show the concentrations of caffeine (CAF) (A-1, B-1, C-1, D-1), chlorogenic acids (CGA) (A-2, B-2, C-2, D-2), trigonelline (TG) (A-3, B-3, C-3, D-3), reducing sugars (RS) (A-4, B-4, C-4, D-4). CGA, RS, and TG decreased progressively with increasing roast level, whereas CAF showed minimal dependence on roast level and consistently remained higher in Robusta than in Arabica. (For interpretation of the references to color in this figure legend, the reader is referred to the web version of this article.)

Across both species, CGA decreased progressively with increasing roast level (Fig. 2A), consistent with its thermal degradation and transformation into downstream phenolic products (e.g., caffeic acid- and quinic acid-related derivatives). RS declined markedly with roasting (Fig. 2B), reflecting the consumption of sugars as key reactants in Maillard reactions and caramelization processes that drive flavor and color development. TG also decreased with increasing roast level (Fig. 2C), consistent with progressive thermal degradation and conversion to niacin (nicotinic acid) and related products. Meanwhile, CAF exhibited comparatively minor roast dependence (Fig. 2D). Robusta consistently exhibited higher CAF levels than Arabica, reflecting intrinsic species-dependent compositional differences. A slight apparent increase in CAF at darker roasts may partially result from roast-induced mass loss (e.g., moisture and volatiles), which increases the concentration on a mass-normalized basis.

### 3.4 Global roasting trends and species differences

To evaluate roasting effects across the entire dataset, the relationships between roast level (Agtron) and compound concentrations were assessed using regression analysis (Fig. 3). Notably, higher Agtron values correspond to lighter roasts, whereas lower values indicate darker roasts.

**Fig. 3:**
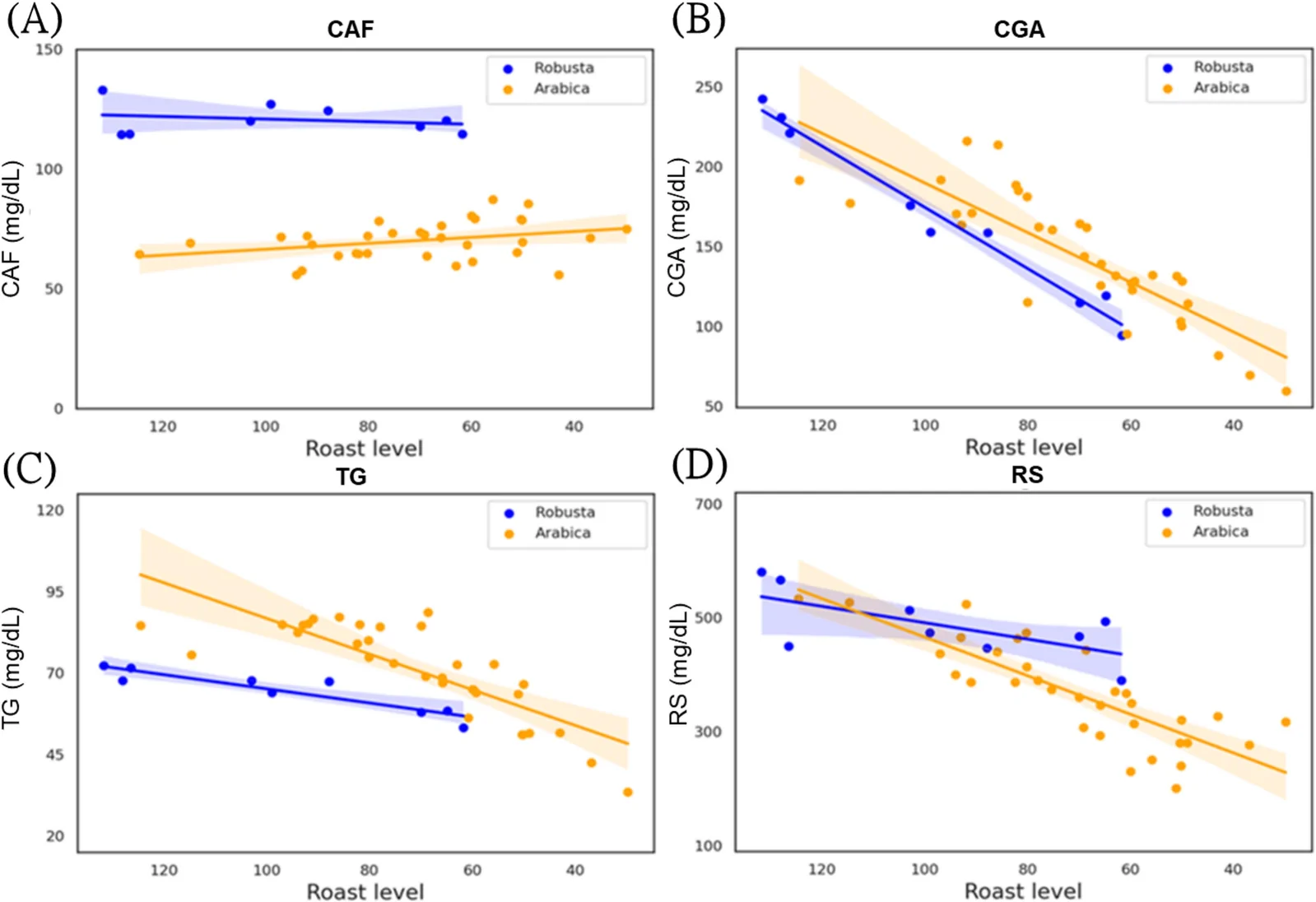
Species-dependent regression relationships between roast level and electrochemically quantified coffee constituents. Scatter plots show the concentrations of (A) CAF, (B) CGA, (C) TG, and (D) RS as functions of roast level (Agtron) for the complete dataset of Arabica and Robusta samples. Solid lines represent linear regression fits with shaded confidence intervals. Higher roast-level values correspond to lighter roasts, whereas lower values indicate darker roasts. CGA, TG, and RS showed positive associations with numerical Agtron values, corresponding to progressive depletion as roast intensity increased.

Consistent with the representative trajectories in Fig. 2, CGA, TG, and RS showed positive associations with numerical Agtron values (Figs. 3B–D), corresponding to progressive depletion as roast intensity increased. Meanwhile, CAF exhibited a comparatively weak dependence on roast level (Fig. 3A). Species-specific differences were evident: Robusta maintained higher CAF concentrations, whereas Arabica generally showed higher TG levels; CGA and RS decreased with roast intensity in both species. These trends align with established roasting chemistry, in which lighter roasts preserve higher levels of native bioactive compounds (e.g., CGA and TG) whereas darker roasts promote degradation and formation of roasted, caramelized, and bitter-tasting products [Laukaleja & Kruma, 2019]. The species-dependent compositional trends observed here are also consistent with previous chromatographic studies. Ky et al. [2001] reported higher CAF and CGA concentrations in Robusta and higher TG and sugar contents in Arabica, while Ogutu et al. [2022] similarly demonstrated substantial species-dependent variation in these constituents. The present electrochemical measurements reproduce these established compositional tendencies using a portable sensing platform, supporting the chemical plausibility of the extracted features.

The combined electrochemical features, CAF, CGA, TG, and RS, together with roast level, provide a chemically interpretable feature set that can support discrimination between Arabica and Robusta and serve as the basis for subsequent ML classification and blend-ratio prediction.

### 3.5 ML-based classification of coffee species and blends

ML models were employed to classify coffee samples as Arabica, Robusta, or blended based on electrochemical and roasting features. RF and XGBoost classifiers were trained using five variables: CAF, CGA, TG, RS, and roast level. Both models demonstrated excellent classification performance (Fig. 4). The RF classifier achieved an overall test accuracy of 97.37%, whereas XGBoost correctly classified all test samples, yielding an accuracy of 100% (Fig. 4C, E). Because the dataset was moderately imbalanced, classification performance was further evaluated using class-specific precision, recall, and F1-score (Table S9A). For RF, the Robusta class achieved precision, recall, and F1-score values of 1.000, indicating that performance for the minority class was not compromised by class imbalance. The corresponding macro-averaged precision, recall, and F1-score were 0.981, 0.979, and 0.980, respectively. XGBoost achieved precision, recall, and F1-score values of 1.000 for all three classes.

**Fig. 4:**
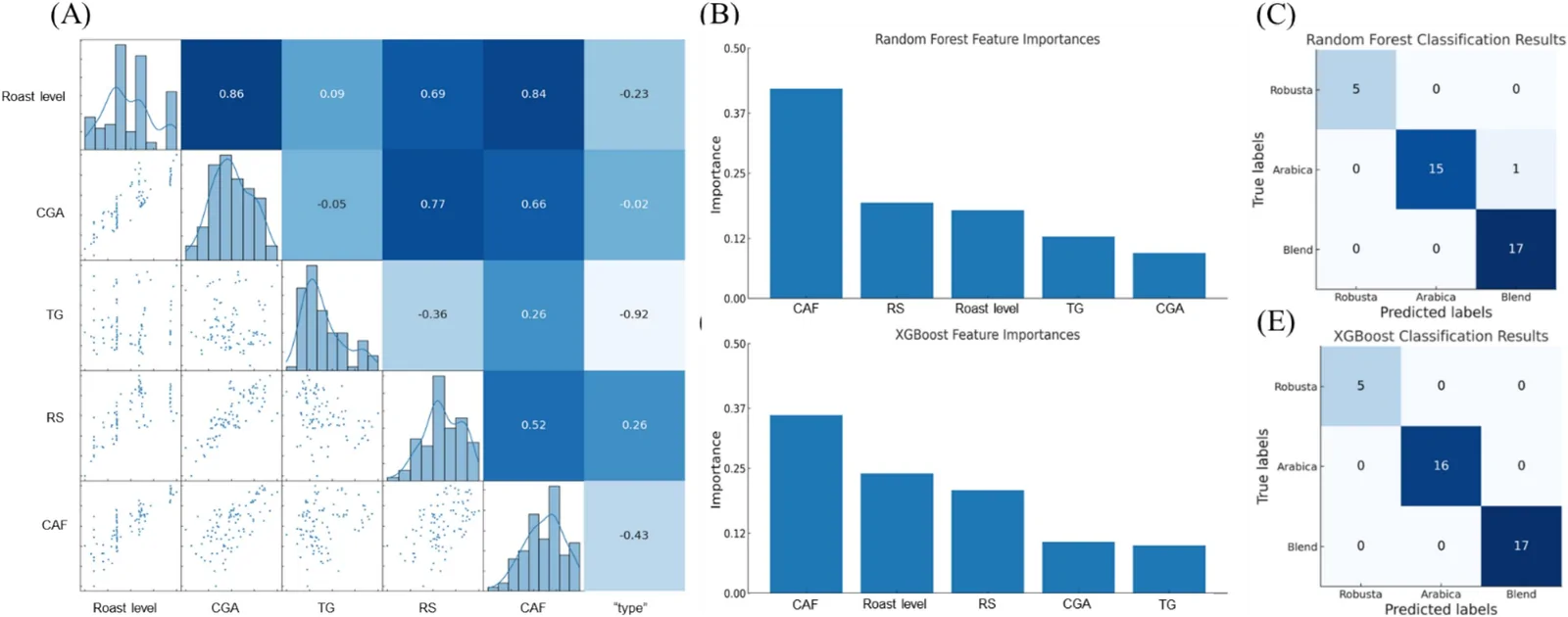
Multivariate electrochemical features enable accurate ML classification of Arabica, Robusta, and blended coffees. (A) Pairwise relationships and Pearson correlation coefficients among the five input variables: roast level, CGA, TG, RS, and CAF. (B) RF feature importance estimated via Gini impurity. (C) RF classification performance on the test set, presented as a confusion matrix. (D) XGBoost feature importance estimated via gain. (E) XGBoost classification performance on the test set, presented as a confusion matrix.

Recent sensing–ML studies have similarly demonstrated strong coffee-authentication performance. For example, Malavi et al. [2026] combined NIR hyperspectral imaging with ML for Robusta adulteration detection and quantification, achieving external-test balanced accuracies above 98%; however, direct numerical comparison with the present study should be made cautiously because of differences in sensing modality, sample composition, and validation design.

Feature importance analysis (Fig. 4B and D) revealed that CAF concentration was the most influential variable for species discrimination, followed by CGAs and roast level. However, using CAF as the sole input feature led to substantial misclassification between Arabica and blended samples, indicating that single-variable classification is insufficient. The integration of multiple electrochemical features markedly improved classification accuracy, demonstrating that the combined compositional fingerprint of CAF, CGA, TG, RS, and roast level provides robust discrimination of coffee species and blends.

These results indicate that electrochemical fingerprints obtained from portable sensing devices effectively capture the compositional differences between the coffee species, enabling reliable ML-based authentication. Both models achieve high classification accuracy using the combined electrochemical profile. CAF was the strongest discriminating variable, whereas incorporating multiple chemical and roasting descriptors improved the separation between Arabica and blended samples.

To assess whether species discrimination was retained for previously unseen origins, a separate leave-one-origin-out analysis was conducted using only pure Arabica and Robusta samples. Under this stricter origin-level validation, accuracy, balanced accuracy, and macro-F1 were all 1.000, with class-specific recall of 1.000 for both species. Because this analysis excludes the blend class, it represents an origin-generalization test of pure-bean species discrimination rather than a direct replication of the original three-class classification task. The classification performance under the different validation strategies is summarized in Table S9B.

Previous studies have demonstrated Arabica/Robusta discrimination using spectroscopic or mass-spectrometric fingerprints. For example, Sahachairungrueng et al. [2022] used NIR hyperspectral imaging and FTIR spectroscopy, Tsai et al. [2023] applied single-bean mass spectrometry with ML, and Gomes et al. [2022] combined multispectral imaging with ensemble learning. In contrast, the present approach relies on a compact set of chemically interpretable electrochemical descriptors together with roast level. Although direct numerical comparison of classification accuracy across studies is not appropriate because of differences in sample composition and validation design, these results demonstrate that portable electrochemical fingerprints can provide an alternative analytical basis for coffee authentication.

### 3.6 Prediction of blend ratios using ANN

To quantitatively estimate the proportion of Arabica and Robusta in blended coffees, an ANN regression model was developed using the same electrochemical feature set. The ANN model demonstrated excellent predictive performance. On the training dataset, the model achieved R^2^ = 0.995, RMSE = 0.024, and MAE = 0.016. On the held-out random-split test set, prediction accuracy remained high with R^2^ = 0.980, RMSE = 0.050, and MAE = 0.032 (Table 2).

**Table 2:** Comparison of test results from different models on coffee blend ratio prediction.

	Dataset	R^2^	RMSE	MAE
Multiple linear regression	**Training set**	0.127	0.329	0.310
	**Test set**	0.146	0.328	0.306
RF regression	**Training set**	0.991	0.033	0.017
	**Test set**	0.951	0.079	0.043
ANN	**Training set**	0.995	0.024	0.016
	**Test set**	0.980	0.050	0.032

Because the random split can distribute samples derived from the same Arabica–Robusta pairing across both the training and test subsets, these results are interpreted here as a within-distribution benchmark rather than as a definitive estimate of generalization to previously unseen blend combinations. To provide a more conservative assessment, additional group-aware evaluations were performed using the nested ANN ensemble framework described in Section 2.3.4. The results of the progressively stricter validation analyses are summarized in Fig. 5. The detailed performance of the original random-split benchmark, including training and test parity plots, tolerance-based prediction performance across blend levels, the corresponding confusion matrix, and training characteristics, is provided in Fig. S6.

**Fig. 5:**
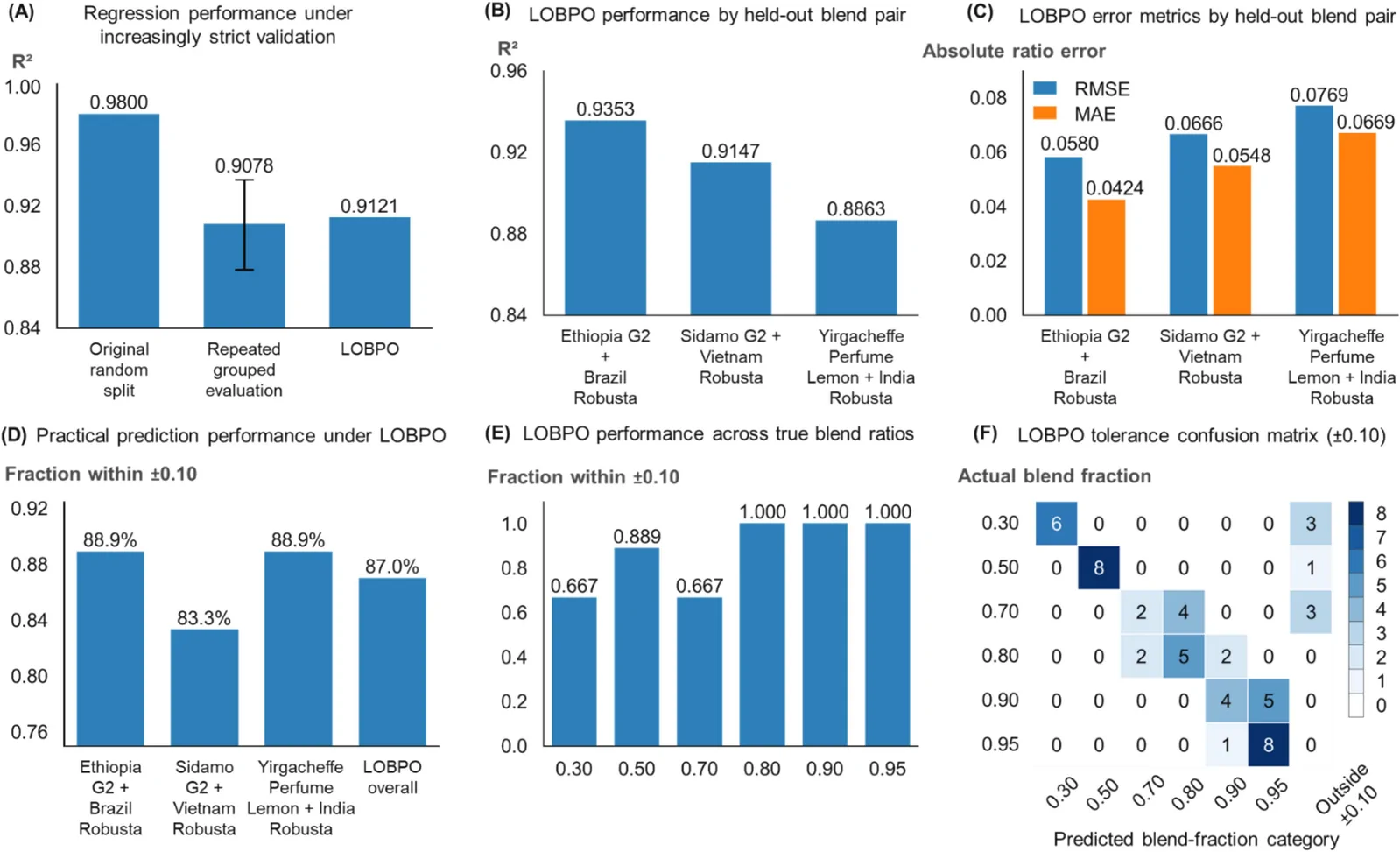
Group-aware validation of the ANN ensemble for Arabica–Robusta blend-ratio prediction. (A) Comparison of regression performance under the original random split, repeated grouped evaluation, and leave-one-blend-pair-out (LOBPO) validation. Error bars for repeated grouped evaluation represent the standard deviation arising from repeated stochastic ANN refitting. (B) Pair-specific R^2^ values obtained when each Arabica–Robusta blend pair was completely excluded from model development. (C) Corresponding RMSE and MAE values for each held-out blend pair. (D) Fraction of predictions falling within ±0.10 of the reference blend fraction for each held-out pair and for the pooled LOBPO predictions. (E) Prediction performance within ±0.10 at individual true blend fractions. (F) Tolerance confusion matrix for the pooled LOBPO predictions; predictions differing from the reference value by more than ±0.10 are assigned to the “Outside ±0.10” category. All regression metrics were calculated using the original continuous ANN outputs without nearest-label snapping or post-hoc calibration.

Under repeated grouped evaluation, in which the same three unique blend-pair partitions were repeatedly refitted to quantify stochastic model variability, the ANN ensemble yielded R^2^ = 0.9078 ± 0.0294, RMSE = 0.0685 ± 0.0106, and MAE = 0.0559 ± 0.0133. The corresponding fraction of predictions within ±0.10 of the reference blend ratio was 0.8241 ± 0.0610. These values were lower than those obtained from the random split, as expected under the stricter group-aware partitioning, but remained consistent across repeated ANN refits.

A stricter leave-one-blend-pair-out (LOBPO) analysis was then performed to evaluate generalization to blend combinations that were completely absent from model development. In each outer fold, all 18 samples derived from one Arabica–Robusta pairing were withheld together, including all six experimentally prepared blend ratios, while the remaining samples were used for scaling, inner model selection, ensemble construction, and model fitting. Across the three outer folds, pooled LOBPO predictions yielded R^2^ = 0.9121, RMSE = 0.0676, and MAE = 0.0547. Furthermore, 47 of the 54 held-out predictions (87.04%) were within ±0.10 of the reference blend fraction.

The pair-specific results further revealed moderate variation among previously unseen blend combinations. When Ethiopia G2 + Brazil Robusta was held out, the model achieved R^2^ = 0.9353, RMSE = 0.0580, and MAE = 0.0424. Holding out Sidamo G2 + Vietnam Robusta yielded R^2^ = 0.9147, RMSE = 0.0666, and MAE = 0.0548. The most challenging pair was Yirgacheffe Perfume Lemon + India Robusta, for which (R^2^ = 0.8863), RMSE = 0.0769, and MAE = 0.0669. Nevertheless, 16 of 18 predictions for this pair (88.9%) remained within the ±0.10 tolerance.

Inspection of the Yirgacheffe Perfume Lemon + India Robusta fold indicated that the larger deviations were concentrated at the low blend-ratio end rather than occurring uniformly across the full compositional range. The two predictions exceeding ±0.10 both corresponded to a true blend fraction of 0.30 and were overestimated at approximately 0.417 and 0.455. This pattern is consistent with a pair-specific feature-distribution shift and modest compression of the predicted range. Because the present dataset does not establish a causal relationship with any single chemical constituent, this result is interpreted conservatively as evidence of pair-dependent domain variation rather than as a specific compositional mechanism.

Taken together, the random-split and group-aware results provide complementary information. The original random split demonstrates strong within-distribution predictive capability, whereas the LOBPO analysis provides a more conservative estimate of performance when an entire blend pairing is absent from model development. The decrease from R^2^ = 0.980 under random splitting to R^2^ = 0.9121 under LOBPO indicates that the random split indeed provided an optimistic estimate of generalization, while the retained performance under complete blend-pair holdout supports the transferability of the multivariate electrochemical fingerprints across unseen blend combinations.

An additional limitation of the present regression framework is that the blended samples were prepared using Arabica and Robusta components with comparable roast levels. This design was intentionally adopted to reduce roast-related confounding and to isolate the effects of species composition and blend ratio. However, commercial coffee products may contain beans roasted to substantially different degrees. Under such conditions, a single Agtron value may not adequately represent the roasting history of the individual components, and the resulting CAF, CGA, TG, and RS profiles may not follow the relationships learned from the present matched-roast dataset. Therefore, the current model should be interpreted as validated primarily for blends prepared from components with similar roast levels. Future studies should systematically evaluate mixed-roast blends and investigate roast descriptors that can better capture roast heterogeneity before extending the model to more complex commercial formulations.

To further interpret the ANN model used for blend-ratio prediction, the SHAP summary analysis is shown in Fig. S4, while representative sample-level waterfall and force plots are provided in Fig. S5. The SHAP summary plot indicates that CAF and TG are the most influential variables driving the model output, whereas CGA, RS, and roast level make smaller but still systematic contributions. In general, the SHAP results indicate that the model uses the combined electrochemical and roasting descriptors rather than relying on a single dominant variable.

Because the final regression framework is a nested group-aware ANN ensemble rather than a single fixed ANN, the SHAP analysis was interpreted as an explanation of the representative ensemble prediction framework. Feature contributions were therefore used to characterize the direction and relative importance of the input variables across the ANN-based prediction process rather than to assign mechanistic significance to one specific network architecture.

Overall, the combined regression and interpretability analyses show that the portable electrochemical measurements can be translated into quantitative estimates of Arabica–Robusta blend composition. Importantly, the stricter group-aware analyses demonstrate that predictive performance is reduced but remains substantial when complete blend pairings are excluded from model development, while the observed pair-to-pair variability highlights the need for broader sampling of coffee origins and blend combinations in future validation studies. The use of ANN-based regression is also consistent with our previous electrochemical study, in which ANN-assisted calibration improved the robustness of CAF and CGA sensing under sensor-batch and operational variability [Gu et al., 2023]. The present study extends this concept from analyte calibration to multivariate blend-ratio prediction and further evaluates generalization using group-aware validation.

### 3.7 Comparison with alternative regression models

To benchmark the ANN regression model, two alternative approaches, RF regression and MLR, were evaluated using the same set of input variables (Table 2). The MLR model exhibited poor predictive performance, yielding R^2^ = 0.146, RMSE = 0.328, and MAE = 0.306 on the test dataset. This result indicates that a simple linear combination of variables cannot adequately describe the relationship among coffee composition, roast level, and blend ratio. The RF regression substantially improved predictive accuracy (R^2^ = 0.951, RMSE = 0.079, MAE = 0.043), demonstrating its ability to capture nonlinear relationships between electrochemical features and blend composition. However, RF predictions remain piecewise and may exhibit discontinuities due to the tree-based structure.

Meanwhile, the ANN model achieved the best predictive performance (R^2^ = 0.980, RMSE = 0.050, MAE = 0.032) because its multilayer architecture can effectively learn complex nonlinear interactions among chemical constituents and roasting conditions. These results indicate that the ANN model provides the most suitable framework for modeling the nonlinear chemical relationships governing coffee blending ratios.

### 3.8 External validation using literature-reported datasets

To further evaluate model generalizability, compositional data from previously published coffee studies were extracted [Šeremet et al., 2022; Heo et al., 2020] and used as external inputs to the trained classification model. The dataset included 17 Arabica and 3 Robusta samples, each with reported concentrations of CAF, CGAs, TG, and sugar-related compositional data. Using the trained XGBoost classification model, all external samples were correctly identified, yielding 100% classification accuracy (Table 3).

**Table 3:** Classification results based on literature data [Šeremet et al., 2022; Heo et al., 2020].

	Predicted Arabica	Predicted Robusta
Arabica	**17**	**0**
Robusta	**0**	**3**

This literature-based validation was restricted to species classification. We were unable to identify a published blended-coffee dataset that simultaneously reported a known Arabica/Robusta blend ratio together with all five variables required by the regression model (CAF, CGA, TG, RS, and roast level). Because imputing or reconstructing missing variables would not constitute an independent external validation, external validation of blend-ratio prediction was not performed. Instead, generalization of the regression framework was assessed separately using the nested group-aware LOBPO procedure described in Section 3.6. Nevertheless, validation using independently collected blended coffees from additional origins and blend combinations remains necessary before broader application. The performance under progressively stricter validation strategies is summarized in Table S10, and the corresponding pair-specific LOBPO results are provided in Table S11. For additional visualization of the direction of prediction errors, the pooled continuous LOBPO predictions were mapped to the nearest nominal blend-ratio labels in Fig. S8; this mapping was used only for visualization and not for calculation of regression metrics.

## 4 Conclusion

This study presents a portable analytical framework that integrates **electrochemical sensing** with **ML** for the rapid authentication of coffee species and the estimation of blend composition. Using a microliter-scale workflow, electrochemical measurements of **CAF, CGAs, TG, and RS**, together with **roast level**, generated informative compositional fingerprints that captured both **species-dependent differences between Arabica and Robusta** and **roast-related chemical transformations**.

For qualitative authentication, the optimized **XGBoost** classifier achieved **100% accuracy** in discriminating among **Arabica, Robusta, and blended** samples. For quantitative assessment, an **ANN regression** model predicted Arabica–Robusta blend ratios with high accuracy (**test R**^**2**^ **= 0.980, RMSE = 0.050, MAE = 0.032**). The original random-split ANN regression yielded R^2^ = 0.980, whereas the more stringent nested group-aware LOBPO evaluation yielded R^2^ = 0.9121, RMSE = 0.0676, and MAE = 0.0547. The modest decrease under complete blend-pair holdout confirms that the random split provided an optimistic within-distribution estimate, while the LOBPO results provide a more conservative assessment of generalization to previously unseen blend combinations. Pair-specific R^2^ values ranged from 0.8863 to 0.9353, indicating that broader sampling across origins and blend combinations will be important before wider deployment. In addition, external validation using **compositional datasets** reported in the literature confirmed the **classification model's transferability**, achieving **100% correct identification** of Arabica and Robusta samples. Although the literature-based external dataset supported species classification, independent external validation of blend-ratio regression could not be performed because no suitable published blend dataset contained all required chemical and roast-level variables. Future studies should therefore validate the regression framework using independently collected blended-coffee samples from additional origins and processing conditions.

The proposed electrochemical–ML platform requires a small aliquot of coffee extract and enables electrochemical measurements within 3 min after sample preparation, while remaining compatible with portable and potentially low-cost analysis. The total sample-to-result time is longer because coffee grinding, brewing, standing, and filtration are required before electrochemical measurement. These advantages make the method promising for **real-time quality control**, **adulteration detection,** and routine screening in coffee production and retail settings. Overall, this study demonstrates that integrating portable electrochemical measurements with ML can considerably enhance coffee authentication speed and practicality. The proposed framework provides a scalable foundation for intelligent food-quality assessment. It could be extended to other **food and beverage authentication** applications by expanding the range of target analytes and incorporating greater sample diversity. Future studies should expand sampling to include additional origins, processing methods, and harvest seasons as well as evaluate model performance under real-world operational variability (e.g., roast profiling and brewing conditions). Incorporating complementary nondestructive features (e.g., bean surface imaging) may further improve robustness and applicability. Future validation should also include Arabica–Robusta blends prepared from components with different roast levels to determine whether the present roast-level descriptor and electrochemical feature set remain adequate under more heterogeneous commercial blending conditions.

## Declaration of generative AI and AI-assisted technologies

During the preparation of this work, the authors used OpenAI Codex (OpenAI) to assist with English-language revision. All experimental data, clinical classifications, analytical thresholds, and scientific interpretations were supplied or determined by the authors. The authors reviewed and edited all AI-assisted output, verified the reported values against the source records, and take full responsibility for the accuracy, integrity, and content of the publication.

## CRediT authorship contribution statement

**Bing-Chen Gu:** Writing – review & editing, Writing – original draft, Validation, Methodology, Investigation, Formal analysis, Data curation. **Kuan-Jung Chung:** Validation, Methodology, Conceptualization. **Zhen-Wei Hong:** Writing – original draft, Validation, Investigation, Formal analysis. **Ning-Hsieh Yang:** Writing – original draft, Investigation, Formal analysis. **Chia-Che Wu:** Writing – review & editing, Writing – original draft, Validation, Supervision, Project administration, Methodology, Funding acquisition, Conceptualization.

## Funding Source

This research was supported in part by the National Science and Technology Council of Taiwan under grant numbers 112–2221-E-005-087, 113–2622-E-005-005, 113–2221-E-005-058, 114–2221-E-005-047-MY2, and 115–2622-E-005-011. Additional support was provided by the Research and Development Working Circle under Project No. 115TOP2–4 of the Taiwan Comprehensive University System (TCUS), Taiwan.

## Declaration of competing interest

The authors declare that they have no known competing financial interests or personal relationships that could have appeared to influence the work reported in this paper.

## Data Availability

The data supporting the findings of this study are available from the corresponding author upon reasonable request.

## Glossary

Arabica: *Coffea Arabica*
Robusta: *Coffea canephora*
CGA: chlorogenic acids
TG: trigonelline
RS: reducing sugars
TDS: total dissolved solids
WE/ CE/ RE: working electrode/ counter electrode/ reference electrode
Ag/AgCl: silver/silver chloride
LSV: linear sweep voltammetry
DPV: differential pulse voltammetry
ML: ML
RF: Random Forest
XGBoost: extreme gradient boosting
ANN: artificial neural network
R^2^: coefficient of determination
RMSE: root mean square error
MAE: mean absolute error (MAE).
