# Supplementary material for: Portable electrochemical sensing coupled with machine learning for rapid Arabica and Robusta coffee authentication and blend ratio prediction
Source: Food Chem X. 2026 Sep 26;39:104523. doi: 10.1016/j.fochx.2026.104523 (PMC13636340; doi:10.1016/j.fochx.2026.104523)
Supplement: Supplementary file 1 [file mmc1.docx]

Supplementary Information

for

**Portable electrochemical sensing coupled with machine learning for rapid Arabica and Robusta coffee authentication and blend ratio prediction**

**Bing-Chen Gu^a^, Kuan-Jung Chung^b^, Zhen-Wei Hong^c^, Ning-Hsieh Yang^c^, Chia-Che Wu^a,c,d*^**

**by**

**^a^ Graduate Program in Semiconductor and Green Technology, Academy of Circular Economy, National Chung Hsing University, Nantou County, Taiwan**

**^b^ Department of Mechatronics Engineering, National Changhua University of Education, Changhua, Taiwan**

**^c^ Department of Electrical Engineering, National Chung Hsing University, Taichung, Taiwan**

**^d^ i-Center for Advanced Science and Technology, National Chung Hsing University, Taichung, Taiwan**

A full-length manuscript submitted for publication in the Journal of

**Submitted to Food Chemistry: X**

Submitted on May 04, 2026

^*^Corresponding author at: Professor, Department of Mechanical Engineering, National Chung Hsing University, Taichung, 40227, TAIWAN; Email: [josephwu@dragon.nchu.edu.tw](mailto:josephwu@dragon.nchu.edu.tw)

Fig. S1. Supporting information for the experimental platform, electrochemical detection chemistry, and ML architectures employed in this study. (A) Coffee roast-level analyzer used for Agtron measurements. (B) Portable electrochemical analyzer with a disposable screen-printed sensor chip. (C) Electrochemical oxidation pathway of CAF. (D) Electrochemical oxidation pathway of CGAs. (E) Alkaline hydrolysis of TG to nicotinic acid before electrochemical detection. (F) RS detection via the reduction of Cu²⁺ to Cu₂O under alkaline conditions. (G) Schematic illustration of the RF ensemble model comprising multiple decision trees. (H) XGBoost model architecture employing sequentially boosted trees. (I) Representative ANN base architecture used within the nested group-aware ANN ensemble framework for blend-ratio regression. Multiple ANN architectures were evaluated within each outer training fold, and four selected ANN members were combined using fixed rank-based ensemble weights for the final continuous blend-ratio prediction.

**Fig. S2. Validation strategies used for machine-learning model evaluation.** (A) Conventional 10-fold cross-validation procedure used to assess model stability under the original within-distribution setting. The training dataset was divided into ten folds, with one fold used for validation in each iteration and the remaining nine folds used for training. (B) Nested group-aware ANN ensemble validation workflow for Arabica–Robusta blend-ratio regression. In each outer leave-one-blend-pair-out (LOBPO) fold, all 18 samples belonging to one complete Arabica–Robusta blend pair were withheld from model development. Min–Max scaling was fitted exclusively using the outer-training data. Candidate ANN models were evaluated using a dual-inner consensus strategy combining pseudo-LOBPO and repeated stratified inner validation. Four unique ANN architectures were selected and combined using fixed rank-based ensemble weights. The held-out outer-test samples were used only for final evaluation, and no reference normalization, post-hoc calibration, or nearest-label snapping was applied.

**Table S1. Model settings and search space used for classification and nested group-aware ANN regression.**

| **Model** | **Task / component** | **Parameter** | **Setting or search range** |
| --- | --- | --- | --- |
| Random Forest (scikit-learn) | Classification | n_estimators | 300 |
|  |  | max_depth | None |
|  |  | min_samples_split | 2 |
|  |  | min_samples_leaf | 1 |
|  |  | random_state | 42 |
|  |  | oob_score | Enabled |
| XGBoost | Classification | n_estimators | 400 |
|  |  | learning_rate | 0.05 |
|  |  | max_depth | 4 |
|  |  | subsample | 0.8 |
|  |  | colsample_bytree | 0.8 |
|  |  | random_state | 42 |
| ANN ensemble (PyTorch) | Blend-ratio regression | Input features | Roast level, CAF, CGA, TG, RS |
|  | Candidate pool | Base architectures | 16 ANN architectures |
|  |  | Seeds per base architecture | 3 |
|  |  | Candidates per outer fold | 48 |
|  | Architecture search | Hidden-layer configurations | 500; 500–128; 512–256; 384–192; 256–128 |
|  |  | Model variants | Regularized, noise-augmented, balanced, blend-focused, and multi-task |
|  | Optimization | Activation | ReLU6 |
|  |  | Primary loss | Huber |
|  |  | Optimizer | Adam |
|  |  | Learning rate | $1\times{10}^{-4}-2\times{10}^{-4}$ |
|  |  | Dropout | 0.05–0.10 |
|  |  | Weight decay | $0-1\times{10}^{-5}$ |
|  |  | Batch size | 32 or 35 |
|  | Inner training | Maximum epochs | 1,200 |
|  |  | Early-stopping patience | 90 epochs |
|  |  | LR scheduler | ReduceLROnPlateau |
|  |  | LR-scheduler patience | 30 epochs |
|  |  | LR reduction factor | 0.5 |
|  |  | Minimum learning rate | $1\times{10}^{-6}$ |
|  | Inner validation | Pseudo-LOBPO | 2 directions; one complete blend pair withheld |
|  |  | Repeated stratified validation | 3 splits; validation fraction = 0.25 |
|  | Final ensemble | No. of selected architectures | 4 unique ANN architectures |
|  |  | Rank-based weights | 0.35 / 0.25 / 0.25 / 0.15 |
|  | Final refit | Seeds | 7, 37, 73 |
|  |  | Epoch rule | $1.3\times inner-selected epoch$ |
|  |  | Epoch bounds | 120–1,400 |
|  | Preprocessing | Scaling | Min–Max; fitted on outer-training data only |
|  | Output | Prediction | Continuous Arabica blend fraction |
|  | Post-processing | Reference normalization | None |
|  |  | Post-hoc calibration | None |
|  |  | Nearest-label snapping | None |

Note: For the nested group-aware regression analysis, all preprocessing, architecture selection, hyperparameter selection, epoch selection, and ensemble construction were performed exclusively within the corresponding outer-training set. The held-out outer-test samples were not used during model development. Multi-task ANN variants included an auxiliary blend-ratio classification head during training; however, all reported regression metrics were calculated from the continuous ANN regression output.

Fig. S3. Features extracted from voltammetric curves for ML input variables. (A) DPV curve of CAF, showing two characteristic oxidation peaks. Peak currents (CAF_I1, CAF_I2), peak potentials (CAF_V1, CAF_V2), and baseline-corrected peak areas (CAF_A1, CAF_A2) were extracted. (B) LSV and DPV responses of CGAs, from which peak descriptors, including CGA_I1, CGA_V1, and CGA_A1, were determined. (C) Electrochemical response of TG, with its characteristic oxidation peak quantified as TG_I, TG_V, and TG_A. (D) Voltammetric response of RS, where peak current (Sugar_I) and peak potential (Sugar_V) were measured as quantitative features. These extracted features represent physically interpretable electrochemical descriptors used as input variables for ML models.

Section S1. ANN architecture screening and nested ensemble model selection for blend-ratio regression

The ANN regression strategy was developed in two stages. First, preliminary within-distribution experiments were conducted to examine the effects of activation function, loss function, and network capacity using the original random train/test split. Second, the final robustness assessment used a nested group-aware ANN ensemble framework in which architecture selection and model optimization were performed exclusively within each outer training fold.

In the preliminary screening stage, the input feature set was kept constant and comprised CAF, CGA, TG, RS, and roast level. Three activation functions—Softmax, Sigmoid, and ReLU6—were compared under the original random-split setting (Table S2). ReLU6 provided the best overall balance of training and test performance. Three loss functions—MAE, MSE, and Huber—were subsequently evaluated using ReLU6 activation (Table S3). Huber loss produced the most favorable overall regression performance and was therefore retained as the primary loss function for subsequent ANN development.

Network-capacity screening was also performed under the original random-split setting using models with 1,500, 1,000, and 500 hidden units (Table S4). The 1,500-neuron model yielded the highest random-split test performance (R^2^ = 0.980, RMSE = 0.050, and MAE = 0.032), whereas the 500-neuron model retained comparable performance with reduced network size. These experiments were used as preliminary architecture screening and as the basis for defining the candidate ANN search space; they were not used to designate a single fixed architecture for the final group-aware analysis.

To provide a more rigorous assessment of model generalization, the final regression framework used nested leave-one-blend-pair-out (LOBPO) validation. In each outer fold, all 18 samples belonging to one complete Arabica–Robusta blend pair were withheld from model development and used only for final evaluation. The remaining 120 samples constituted the outer-training set. Min–Max scaling was fitted exclusively to the outer-training data and applied unchanged to the corresponding inner-validation and outer-test samples.

Within each outer-training fold, model selection was conducted using a dual-inner consensus strategy comprising two complementary validation procedures. The first was pseudo-LOBPO validation, in which one complete blend pair within the outer-training data was withheld as an inner validation group. The second was repeated stratified inner validation using three independent splits with a validation fraction of 0.25. Candidate models were ranked according to their performance across both inner-validation schemes to reduce dependence on a single data partition.

The ANN candidate pool comprised 16 base architectures evaluated using three random seeds, resulting in 48 candidate models per outer fold. Candidate hidden-layer configurations included 500, 500–128, 512–256, 384–192, and 256–128 neurons. Additional variants incorporated regularization, noise augmentation, balanced weighting, blend-focused weighting, and multi-task learning. All candidate networks used ReLU6 activation and the Adam optimizer, with Huber loss as the primary regression objective. Dropout rates of 0.05–0.10, learning rates of $1\times{10}^{-4}-2\times{10}^{-4}$, weight decay of $0-1\times{10}^{-5}$, and batch sizes of 32 or 35 were evaluated.

Inner-loop training was limited to a maximum of 1,200 epochs and employed early stopping with a patience of 90 epochs. The learning rate was adjusted using ReduceLROnPlateau with a patience of 30 epochs, a reduction factor of 0.5, and a minimum learning rate of $1\times{10}^{-6}$. For each outer fold, four unique ANN architectures showing the most stable consensus performance across the two inner-validation strategies were selected to form the final ensemble. Rank-based ensemble weights of 0.35, 0.25, 0.25, and 0.15 were assigned to the four selected members. Each selected member was subsequently refitted using random seeds 7, 37, and 73. The final training duration was defined as 1.30 times the epoch selected during inner validation, constrained to a range of 120–1,400 epochs.

The final blend-ratio estimate was obtained from the weighted average of the continuous outputs of the selected ANN members. Multi-task ANN variants contained an auxiliary blend-ratio classification head during training, but the final reported prediction remained the continuous regression output. No reference normalization, post-hoc calibration, or nearest-label snapping was applied.

This nested model-selection procedure ensured that the outer held-out blend pair remained completely isolated from preprocessing, architecture selection, hyperparameter tuning, epoch selection, ensemble construction, and model refitting. Accordingly, the resulting LOBPO performance provides a more conservative estimate of prediction for previously unseen Arabica–Robusta blend combinations than the original random-split benchmark. The overall validation workflow is illustrated in Fig. S2B, and the model settings and search ranges are summarized in Table S1.

Section S2. Independent laboratory analyses used for method comparison

Selected coffee samples were independently analyzed by JITTEC Service Corporation (Taiwan) to provide external laboratory comparator measurements for the electrochemical platform.

Caffeine. CAF was determined according to CNS 9432 (N6174), Method of test for caffeine content in foods. The resulting concentrations were used as standardized external comparator values for evaluation of the electrochemical CAF measurements.

Chlorogenic acids and trigonelline. CGA and TG were determined by JITTEC using laboratory-developed HPLC procedures. No official TFDA analytical method specifically applicable to coffee samples was used for these two constituents in the present study. Because the detailed chromatographic conditions and internal standard operating procedures were not included in the analytical results supplied to the authors, information regarding column chemistry, mobile-phase composition, detector configuration, and laboratory calibration procedures is not reported. These measurements were therefore treated as independent laboratory HPLC comparator data rather than as fully reproduced standardized reference methods.

Sugars. Fructose, glucose, sucrose, maltose, and lactose were determined according to CNS 12634 (2006), Method of test for fruit and vegetable juice products – Determination of sugars by HPLC. According to the JITTEC reports, the quantification limit for each individual sugar was 0.2 g/100 mL, and total-sugar concentrations were additionally reported. Because the electrochemical assay measures a reducing-sugar-related response whereas the external method reports individual and total sugars, these results were used as supportive external comparisons rather than as evidence of direct analytical equivalence.

**Table S2. Preliminary comparison of activation functions under the original random-split ANN benchmark.**

| **Activation function** | **Dataset** | **R²** | **RMSE** | **MAE** |
| --- | --- | --- | --- | --- |
| Softmax | Training | 0.922 | 0.099 | 0.033 |
|  | Test | 0.842 | 0.141 | 0.051 |
| Sigmoid | Training | 0.983 | 0.047 | 0.033 |
|  | Test | 0.977 | 0.053 | 0.038 |
| ReLU6 | Training | 0.995 | 0.024 | 0.016 |
|  | Test | 0.980 | 0.050 | 0.032 |

**Table S3. Preliminary comparison of loss functions under the original random-split ANN benchmark (ReLU6 activation).**

| **Loss function** | **Dataset** | **R²** | **RMSE** | **MAE** |
| --- | --- | --- | --- | --- |
| MAE | Training | 0.991 | 0.033 | 0.021 |
|  | Test | 0.975 | 0.056 | 0.035 |
| MSE | Training | 0.995 | 0.026 | 0.017 |
|  | Test | 0.979 | 0.051 | 0.032 |
| Huber | Training | 0.995 | 0.024 | 0.016 |
|  | Test | 0.980 | 0.050 | 0.032 |

**Table S4. Preliminary network-capacity screening under the original random-split ANN benchmark (ReLU6 + Huber).**

| **Neurons** | **Dataset** | **R²** | **RMSE** | **MAE** |
| --- | --- | --- | --- | --- |
| 1,500 | Training | 0.995 | 0.024 | 0.016 |
|  | Test | 0.980 | 0.050 | 0.032 |
| 1,000 | Training | 0.987 | 0.042 | 0.031 |
|  | Test | 0.974 | 0.057 | 0.039 |
| 500 | Training | 0.991 | 0.034 | 0.024 |
|  | Test | 0.974 | 0.058 | 0.037 |

These random-split experiments were used for preliminary architecture screening and did not define a single fixed architecture for the final nested group-aware ANN ensemble.

Table S5. Calibration characteristics, linear ranges, limits of detection, and limits of quantification for the portable electrochemical determination of CAF, CGA, TG, and RS.

| Analyte | Features | Replicates per level | Calibration equation | Linear range  (mg dL⁻¹) | R² | Concentration-domain SD  (mg dL⁻¹) | LOD  (mg dL⁻¹) | LOQ  (mg dL⁻¹) |
| --- | --- | --- | --- | --- | --- | --- | --- | --- |
| CAF | CAF-I₂ | n=3 | y=16.70C+171.80 | 20–500 | 0.97 | $\sigma_{c}=2.00$ | 6.60 | 20.00 |
| CGA | CGA-I₁ | n=3 | y=3.50C+4.95 | 20–500 | 0.97 | $\sigma_{c}=1.52$ | 5.02 | 15.21 |
| TG | TG_I | n=3 | y=1.19C+9.18 | 10–100 | 0.99 | $\sigma_{c}=0.38$ | 1.26 | 3.83 |
| RS | RS_I | n=3 | y=0.0904C+15.21 | 65–1250 | 0.95 | $\sigma_{c}=5.84$ | 19.27 | 58.40 |

Table S6. Recovery and matrix-effect results

S6A. Standard-addition recoveries of TG and RS in representative coffee matrices (n=3)

| Analyte | Samples | Species | Roast  (Agtron) | Native concentration  (mg dL^-1^) | Added concentration  (mg dL^-1^) | Measured concentration  (mg dL^-1^) | Recovery (%) | RSD (%) |
| --- | --- | --- | --- | --- | --- | --- | --- | --- |
| TG | Ethiopia G2 | Arabica | 80.2 | 74.77 | 20 | 96.83 | 110.3 | 3.59 |
| TG | Ethiopia G2 | Arabica | 36.9 | 42.38 | 30 | 74.54 | 107.2 | 3.13 |
| TG | Vietnam | Robusta | 103 | 67.61 | 20 | 87.39 | 98.9 | 2.79 |
| TG | Vietnam | Robusta | 64.9 | 58.36 | 30 | 87.52 | 97.2 | 1.35 |
| RS | Ethiopia G2 | Arabica | 80.2 | 413.23 | 100 | 506.13 | 92.9 | 4.23 |
| RS | Ethiopia G2 | Arabica | 36.9 | 276.67 | 200 | 480.87 | 102.1 | 3.98 |
| RS | Vietnam | Robusta | 103 | 513.33 | 100 | 627.23 | 113.9 | 4.57 |
| RS | Vietnam | Robusta | 64.9 | 493.33 | 200 | 682.53 | 94.6 | 3.65 |

Table S6B. Matrix effects on the electrochemical determination of CAF, CGA, TG, and RS in Arabica and Robusta coffee extracts

| Analyte | Matrix | Solvent-calibration slope | Matrix-matched slope | Slope ratio (%) | Signal suppression /enhancement (%) | Interpretation |
| --- | --- | --- | --- | --- | --- | --- |
| CAF | Arabica | 16.70 | 16.75 | 100.3% | +0.3% | Negligible |
| CAF | Robusta | 16.70 | 16.53 | 99.3% | -0.7% | Negligible |
| CGA | Arabica | 3.50 | 3.40 | 97.1% | -2.8% | Negligible |
| CGA | Robusta | 3.50 | 3.71 | 106.0% | +6.0% | Negligible |
| TG | Arabica | 1.19 | 1.21 | 101.7% | +1.7% | Negligible |
| TG | Robusta | 1.19 | 1.17 | 98.3% | -0.7% | Negligible |
| RS | Arabica | 0.0904 | 0.0915 | 101.2% | +1.2% | Negligible |
| RS | Robusta | 0.0904 | 0.0892 | 98.7% | -1.3% | Negligible |

Table S7. Inter-batch reproducibility of the disposable electrochemical platform.

| Analyte | Precision level | No. of measurements | No. of chips | No. of batches | Mean measured concentration (mg dL^-1^) | RSD (%) |
| --- | --- | --- | --- | --- | --- | --- |
| CAF | Inter-batch | 15 | 15 | 3 (5 chips each batch) | 39.7 | 6.23 |
| CGA | Inter-batch | 15 | 15 | 3 (5chips each batch) | 40.5 | 6.76 |
| TG | Inter-batch | 15 | 15 | 3 (5chips each batch) | 61.1 | 6.84 |
| RS | Inter-batch | 15 | 15 | 3 (5chips each batch) | 593.1 | 9.17 |

Table S8A Comparison of electrochemical caffeine measurements with independent laboratory results obtained using CNS 9432.

| Coffee origin | Roast level (Agtron) | Electrochemical CAF  (mg dL^-1^) | JITTEC CAF  (mg dL^-1^) | Absolute difference | Relative difference (%) | External method |
| --- | --- | --- | --- | --- | --- | --- |
| Ethiopia G2 | 80.2 | 72.05 | 77.30 | 5.25 | -6.79% | CNS 9432 |
| Ethiopia G2 | 36.9 | 71.13 | 76.50 | 5.37 | -7.02% | CNS 9432 |
| Robusta Vietnam | 103.0 | 119.97 | 129.10 | 9.13 | -7.07% | CNS 9432 |
| Robusta Vietnam | 64.9 | 120.29 | 128.70 | 8.41 | -6.53% | CNS 9432 |

Table S8B. Comparison of electrochemical CGA and TG measurements with independent laboratory HPLC measurements.

| Analyte | Coffee type / origin | Roast level (Agtron) | Electrochemical result  (mg dL^-1^) | JITTEC HPLC result  (mg dL^-1^) | Absolute difference | Relative difference (%) |
| --- | --- | --- | --- | --- | --- | --- |
| CGA | Ethiopia G2 | 80.2 | 115.12 | 123.35 | 8.23 | -6.67% |
| CGA | Ethiopia G2 | 36.9 | 69.51 | 77.43 | 7.92 | -10.23% |
| CGA | Robusta Vietnam | 103.0 | 175.55 | 183.52 | 7.97 | -4.34% |
| CGA | Robusta Vietnam | 64.9 | 119.95 | 130.4 | 10.45 | -8.01% |
| TG | Ethiopia G2 | 80.2 | 74.77 | 82.21 | 7.44 | -9.05% |
| TG | Ethiopia G2 | 36.9 | 42.38 | 46.32 | 3.94 | -8.51% |
| TG | Robusta Vietnam | 103.0 | 67.61 | 72.23 | 4.62 | -6.40% |
| TG | Robusta Vietnam | 64.9 | 58.36 | 61.23 | 2.87 | -4.69% |

Table S8C. Comparison of electrochemical reducing-sugar measurements with independent sugar-analysis results obtained using CNS 12634 (2006).

| Coffee origin | EC RS  (mg dL^-1^) | Fructose  (mg dL^-1^) | Glucose  (mg dL^-1^) | Sucrose  (mg dL^-1^) | Maltose  (mg dL^-1^) | Lactose  (mg dL^-1^) | Total sugars  (mg dL^-1^) |
| --- | --- | --- | --- | --- | --- | --- | --- |
| Ethiopia G2 | 413.25 | 0 | 500 | 0 | 0 | 0 | 500 |
| Ethiopia G2 | 276.67 | 0 | 200 | 0 | 0 | 0 | **200** |
| Robusta Vietnam | 513.33 | 0 | 200 | 200 | 0 | 0 | **400** |
| Robusta Vietnam | 493.33 | 0 | 400 | 0 | 0 | 0 | 400 |

Table S9. Classification performance under random-split and group-aware validation.

S9A. Class-specific performance on the original random-split test set

| Model | Class | Support | Precision | Recall | F1-score |
| --- | --- | --- | --- | --- | --- |
| RF | Robusta | 5 | 1.000 | 1.000 | 1.000 |
| RF | Arabica | 16 | 1.000 | 0.938 | 0.968 |
| RF | Blend | 17 | 0.944 | 1.000 | 0.971 |
| RF | Macro average | 38 | **0.981** | **0.979** | **0.980** |
| XGBoost | Robusta | 5 | 1.000 | 1.000 | 1.000 |
| XGBoost | Arabica | 16 | 1.000 | 1.000 | 1.000 |
| XGBoost | Blend | 17 | 1.000 | 1.000 | 1.000 |
| XGBoost | Macro average | 38 | **1.000** | **1.000** | **1.000** |

Support denotes the number of true samples of each class in the random-split test set. RF accuracy = 0.9737 and balanced accuracy = 0.9792; XGBoost accuracy and balanced accuracy = 1.000.

S9B. XGBoost classification performance under different data-partitioning strategies

| Validation scheme | Classification task | Accuracy | Balanced accuracy | Macro-F1 | Per-class recall |
| --- | --- | --- | --- | --- | --- |
| Original random split | Arabica/Robusta/Blend | 1.000 | 1.000 | 1.000 | A=1.000; R=1.000; B=1.000 |
| Repeated stratified | Pure Arabica/Robusta | 1.0000 ± 0.0000 | 1.0000 ± 0.0000 | 1.0000 ± 0.0000 | A=1.0000; R=1.0000 |
| Leave-one-origin-out | Pure Arabica/Robusta | 1.000 | 1.000 | 1.000 | A=1.000; R=1.000 |

The random-split analysis is a three-class task including blends, whereas repeated stratified and LOOO analyses evaluate pure-bean Arabica-versus-Robusta discrimination.

Table S10. Regression performance under progressively stricter validation

| Validation scheme | R^2^ | RMSE | MAE | Within ±0.10 |
| --- | --- | --- | --- | --- |
| Original random split | 0.980 | 0.050 | 0.032 | NR |
| Repeated grouped evaluation | 0.9078 ± 0.0294 | 0.0685 ± 0.0106 | 0.0559 ± 0.0133 | 0.8241 ± 0.0610 |
| LOBPO | 0.9121 | 0.0676 | 0.0547 | 0.8704 |

Repeated grouped evaluation contains only three unique blend-pair partitions; repeated runs quantify stochastic ANN initialization and refitting variability rather than additional independent group partitions

Table S11. Pair-specific LOBPO performance

| Held-out pair | R^2^ | RMSE | MAE | Within ±0.10 |
| --- | --- | --- | --- | --- |
| Ethiopia G2 + Brazil Robusta | 0.9353 | 0.0580 | 0.0424 | 88.9% |
| Sidamo G2 + Vietnam Robusta | 0.9147 | 0.0666 | 0.0548 | 83.3% |
| Yirgacheffe Perfume Lemon + India Robusta | 0.8863 | 0.0769 | 0.0669 | 88.9% |

Fig. S4. SHAP interpretation of the ANN blend-ratio model

Fig. S5. (A) SHAP waterfall plot illustrating the contribution of each feature to the model prediction for a representative sample. (B) SHAP force plot visualizing the cumulative push–pull effects of input variables on the predicted blend ratio.

Fig. S6. Within-distribution benchmark performance of the ANN model for Arabica–Robusta blend-ratio prediction under the original random train/test split. (A) Parity plot between predicted and reference blend fractions for the training set. (B) Parity plot for the random-split test set. (C) Fraction of predictions satisfying the predefined ±0.10 tolerance at each blend level. (D) Tolerance-based confusion matrix for the random-split evaluation. (E) Training and validation loss trajectories during ANN optimization. (F) Learning curves showing the evolution of training and validation R^2^. These results represent within-distribution model performance and are provided as a complementary benchmark to the stricter nested group-aware LOBPO validation presented in Fig. 5.

Fig. S7. Calibration curves for the portable electrochemical determination of caffeine (CAF), chlorogenic acids (CGA), trigonelline (TG), and reducing sugars (RS). Calibration relationships between analyte concentration and the corresponding electrochemical response are shown for (A) CAF using the CAF-I_2_ feature, (B) CGA using the CGA-I_1_ feature, (C) TG using the TG_I feature, and (D) RS using the RS_I feature. Each calibration level was measured using three independent replicates, and the data are presented as mean ± standard deviation. Dotted lines represent least-squares linear regression fits. The corresponding regression equations and coefficients of determination (R²) are displayed in each panel. The calibration ranges were 20–500 mg dL^-1^ for CAF and CGA, 10–100 mg dL^-1^ for TG, and 65–1250 mg dL^-1^ for RS.

**Fig. S8. Nearest-label confusion matrix for pooled leave-one-blend-pair-out (LOBPO) predictions of Arabica–Robusta blend ratios.** Continuous ANN predictions from the three outer LOBPO folds were mapped to the nearest nominal blend-ratio label (0.30, 0.50, 0.70, 0.80, 0.90, or 0.95) solely to visualize the direction and proximity of prediction errors. Rows represent the true blend ratios and columns represent the nearest predicted labels. Values in each cell indicate the number of samples assigned to the corresponding predicted ratio. This nearest-label mapping was used only for visualization; all quantitative regression metrics, including R^2^, RMSE, MAE, and the fraction of predictions within ±0.10 of the reference blend fraction, were calculated directly from the original continuous ANN outputs without nearest-label snapping or post-hoc calibration.
